# Nowcasting methods to improve the performance of respiratory sentinel surveillance: lessons from the COVID-19 pandemic

**DOI:** 10.1038/s41598-024-62965-5

**Published:** 2024-05-31

**Authors:** Sandra Flores-Alvarado, María Fernanda Olivares, Natalia Vergara, Christian García, Mauricio Canals, Cristóbal Cuadrado

**Affiliations:** 1 Escuela de Salud Pública, Facultad de Medicina, Universidad de Chile, Av. Independencia 939, Santiago, Chile; 2 Programa de Doctorado en Salud Pública, Escuela de Salud Pública, Facultad de Medicina, Universidad de Chile, Santiago, Chile; 3 Departamento de Epidemiología, Subsecretaría de Salud Pública, Ministerio de Salud de Chile, Santiago, Chile

**Keywords:** SARI, ILI, Emerging diseases, Epidemiological surveillance, Public health, Nowcasting, Respiratory tract diseases, Disease prevention, Epidemiology

## Abstract

Respiratory diseases, including influenza and coronaviruses, pose recurrent global threats. This study delves into the respiratory surveillance systems, focusing on the effectiveness of SARI sentinel surveillance for total and severe cases incidence estimation. Leveraging data from the COVID-19 pandemic in Chile, we examined 2020–2023 data (a 159-week period) comparing census surveillance results of confirmed cases and hospitalizations, with sentinel surveillance. Our analyses revealed a consistent underestimation of total cases and an overestimation of severe cases of sentinel surveillance. To address these limitations, we introduce a nowcasting model, improving the precision and accuracy of incidence estimates. Furthermore, the integration of genomic surveillance data significantly enhances model predictions. While our findings are primarily focused on COVID-19, they have implications for respiratory virus surveillance and early detection of respiratory epidemics. The nowcasting model offers real-time insights into an outbreak for public health decision-making, using the same surveillance data that is routinely collected. This approach enhances preparedness for emerging respiratory diseases by the development of practical solutions with applications in public health.

## Introduction

Throughout the last century, we have witnessed recurrent outbreaks of emerging and re-emerging respiratory diseases, highlighting the influenza virus and coronaviruses as agents with high pandemic potential^1–5^. In 2022, 2 years after the COVID-19 pandemic outbreak, the World Health Organization (WHO) recommended the integration of surveillance for this disease into the sentinel surveillance systems for respiratory diseases^6^. In this context, strong surveillance networks that provide timely, accurate and precise information to detect the signs of emerging epidemics that allow early warnings to be triggered and informed decisions to be made in the management of health crises, are required^4,5^. Data collected during the pandemic represents a unique opportunity to evaluate these capabilities in real-world scenarios, aiming to optimize the use of existing surveillance systems in future epidemics.

Epidemiological surveillance consists of the collection, analysis, interpretation and dissemination of health information for public health actions^7,8^. Surveillance methods focus on being practical, uniform and fast, over their precision or completion, with the purpose of observing changes in trends and taking control measures^9,10^. It is considered the best strategy in the prevention of epidemics, as it allows the timely identification of outbreaks and pathogens, to start containment strategies^8,9^. Globally, surveillance systems for influenza and respiratory diseases are part of the WHO Global Influenza Surveillance and Response System (GISRS) network^11^. The GISRS aims to globally monitor influenza activity and viral circulation, to characterize disease burden and severity, detect risk factors for severe disease and detect unusual activity. To achieve this, countries apply the GISRS surveillance and monitoring recommendations by reporting cases daily at the national level, which include monitoring of outpatient and hospital consultations and deaths due to respiratory causes, as well as sentinel surveillance based on case definitions of influenza-like illness (ILI) and severe acute respiratory infection (SARI)^11^.

Evaluations of sentinel surveillance systems generally focus on processes rather than their performance in adequately estimating the disease incidence^12–14^. The studies who have evaluated these properties conclude that the national and subnational capacities of many countries are insufficient for adequate surveillance, which is reflected in the under-ascertainment and/or under-reporting of cases, especially those asymptomatic or subclinical, insufficient sample sizes, and late and incomplete delivery of information^4,5,15,16^. These elements can lead to problems in detecting epidemic trends and bias in the disease incidence estimation^17^.

The accuracy, precision and timeliness of these estimates can be improved through the implementation of a modeling approach known as nowcasting, which consists of the prediction of current events, or nearby events in the past or future^18^. This strategy allows obtaining valid and reliable diagnoses of the epidemiological situation during an epidemic, even in contexts of lag in the capture and reporting of information, improving the early detection of ongoing outbreaks and facilitating public health decision-making, ultimately favoring the timely implementation of preventive and outbreak control measures^19,20^. Thus, the incorporation of nowcasting in epidemiological surveillance can be an essential strategy to strengthen our surveillance systems and improve decision-making in future epidemiological threats.

In Chile, COVID-19 was integrated into the respiratory disease surveillance system as a novel event with universal and mandatory notification, promptly associated to the digitalization of the notification system and the reinforcement of diagnostic capacities^21,22^. This universal surveillance system allowed the recollection of large volumes of data at the individual level, collected in parallel to influenza sentinel surveillance and to the population registry of hospital beds, which continued to operate during the pandemic and detected respiratory infections corresponding mainly to the SARS-CoV-2 virus^23^. The simultaneous high-quality population level data and sentinel surveillance registries makes Chile a case study of interest to evaluate current epidemiological surveillance strategies of respiratory viruses, comparing the census or population level information of total and severe cases with the existing sentinel surveillance. Likewise, this scenario allows evaluating the feasibility and usefulness of implementing nowcasting strategies based on the usual epidemiological records to improve the precision, accuracy and timeliness of existing sentinel surveillance, which can be replicable in other contexts and for other epidemic agents.

In this context, the objective of this study is two-fold. First, we analyze the capability of SARI sentinel centers to estimate the population incidence of total ILI cases and total severe cases of an emerging epidemic outbreak, accurately and precisely. To achieve this, we compared the population incidences based on census information on existing COVID-19 cases and hospitalizations in Chile, with estimated incidences from SARI sentinel surveillance. Second, we seek to propose a nowcasting model that improves outbreak surveillance capacity, contributing to strengthening the surveillance system and increasing the precision, accuracy, and timeliness of incidence estimates.

## Results

**Figure 1:**
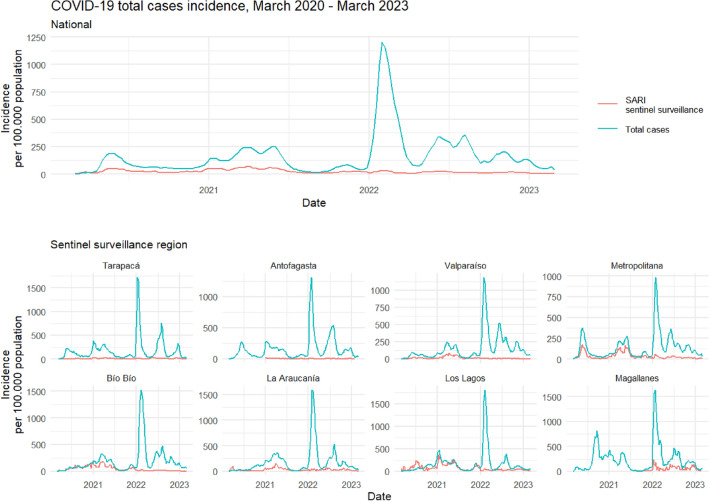
Incidence rate of COVID-19 total cases vs. SARI sentinel surveillance estimates. *SARI* Severe Acute Respiratory Infections. Incidence rate per 100,000 population for the observed national and regional series from March 1st 2020 to March 1st 2023. National series includes only those regions that report for SARI sentinel surveillance. Blue lines represent the observed incidence rate of SARS-CoV-2 total cases (with PCR or antigens test confirmation) from universal surveillance data. Red lines represent the raw estimation of total cases incidence rate from SARI sentinel surveillance data. Antofagasta and Magallanes regions began reporting for SARI sentinel surveillance on 2021 and 2022, respectively.

**Figure 2:**
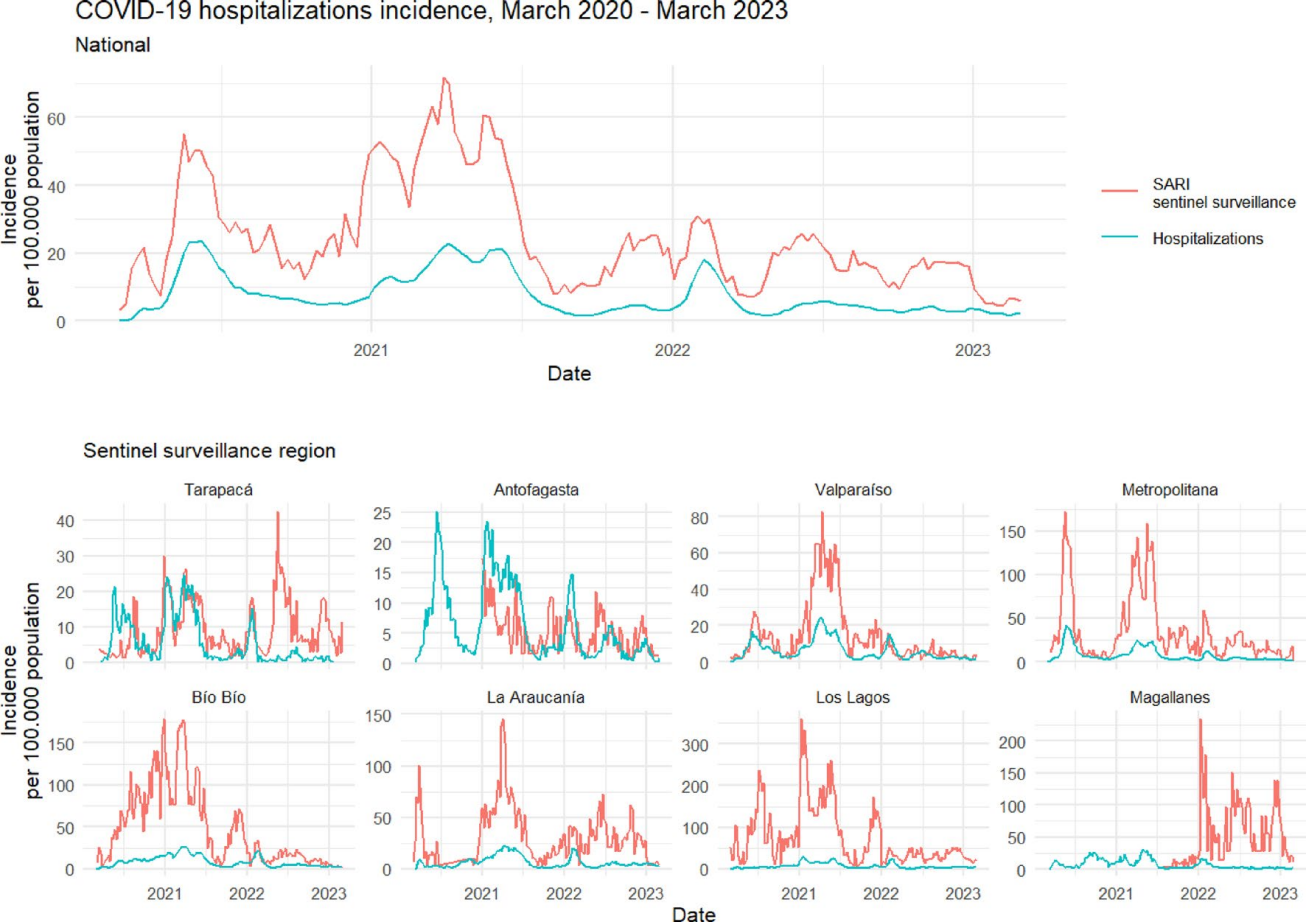
Incidence rate of COVID-19 hospitalizations vs. SARI sentinel surveillance estimates. *SARI* Severe Acute Respiratory Infections. Incidence rate per 100,000 population for the observed national and regional series from March 1st 2020 to March 1st 2023. National series includes only those regions that report for SARI sentinel surveillance. Blue lines represent the observed incidence rate of SARS-CoV-2 hospitalized severe cases (with PCR or antigens test confirmation) from hospital beds census data. Red lines represent the raw estimation of hospitalized severe cases incidence rate from SARI sentinel surveillance data. Antofagasta and Magallanes regions began reporting for SARI sentinel surveillance on 2021 and 2022, respectively.

In this study, we compared the series of total cases (*universal surveillance*, N = 5,024,878), total hospitalized severe cases (*hospital beds*, N = 236,713), and severe cases surveillance (*SARI sentinel surveillance*, n = 15,085) of COVID-19 at the national and regional level in the 8 regions of Chile that are part of the surveillance network, during a period of 159 epidemiological weeks (EW) (EW10-2020–EW09-2023). The results reveal that SARI sentinel surveillance underestimates the incidence of total cases while overestimating severe cases with respect to the census incidence (Figs. 1 and 2). When corrected for risk of hospitalization, total cases are also overestimated (Fig. S1). The pattern is repeated both at the regional level and within different age groups (Figs. S2 and S3).

**Figure 3:**
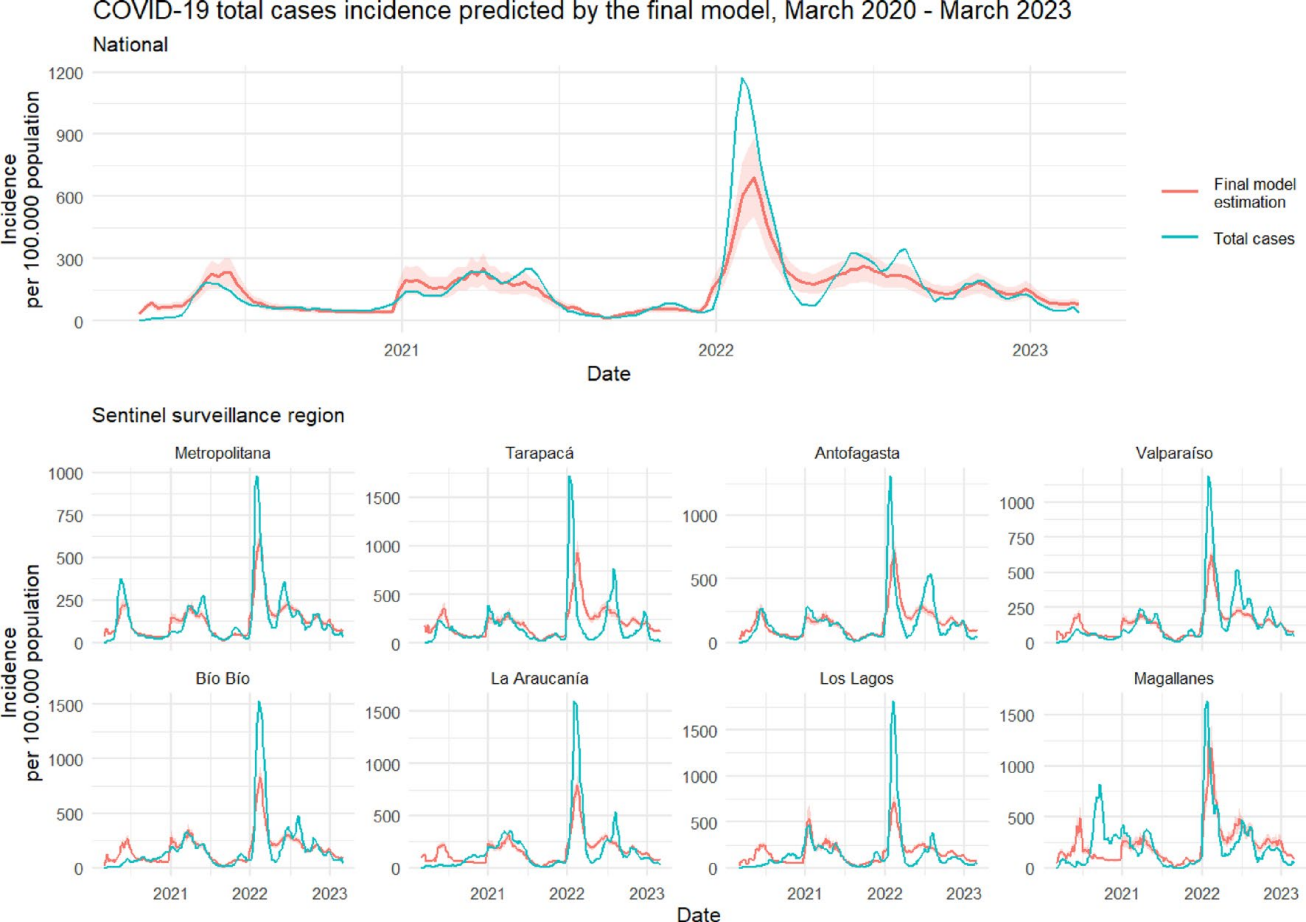
Final model estimation of COVID-19 total cases incidence rate. Incidence rate per 100,000 population for the national and regional series from March 1st 2020 to March 1st 2023. The national series includes data aggregated from all regions. Blue lines represent the observed incidence rate of SARS-CoV-2 total cases (with PCR or antigens test confirmation) from universal surveillance data. Red lines represent the final model (model 3) estimation of total cases incidence rate. Red color bands around red lines represent the 95% confidence intervals of the fitted models.

**Figure 4:**
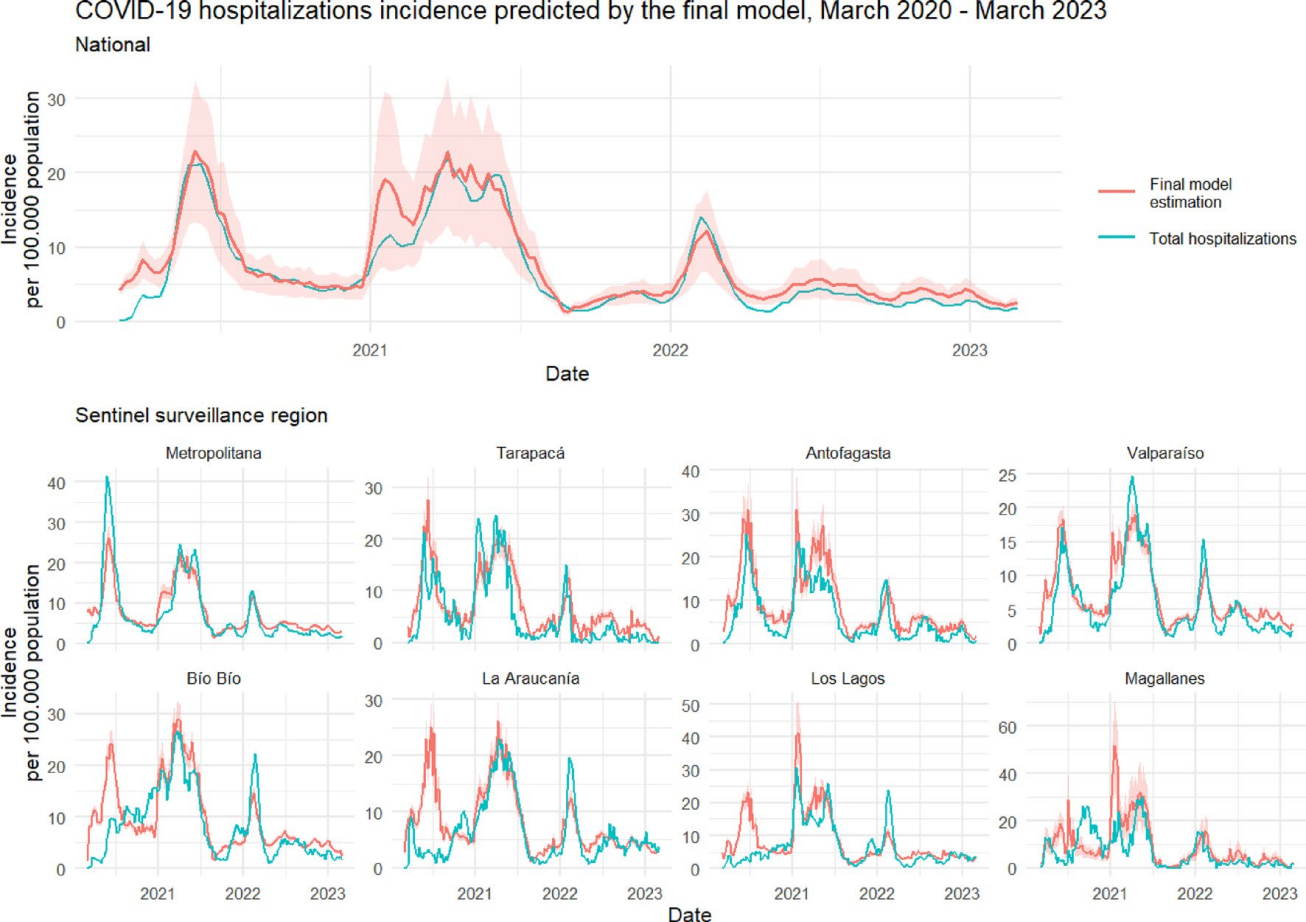
Final model estimation of COVID-19 hospitalization cases incidence rate. Incidence rate per 100.000 population for the national and regional series from March 1st 2020 to March 1st 2023. The national series includes data aggregated from all regions. Blue lines represent the observed incidence rate of SARS-CoV-2 hospitalized severe cases (with PCR or antigens test confirmation) from hospital beds census data. Red lines represent the final model (model 3) estimation of hospitalized severe cases incidence rate. Red color bands around red lines represent the 95% confidence intervals of the fitted models.

Due to the impossibility of directly estimating the population incidence of total and severe cases from sentinel surveillance of respiratory viruses, we fitted a generalized linear model with mixed effects and negative binomial response. We used the incidence rate estimated by SARI sentinel surveillance and other covariates (see “Methods”) to estimate census incidence of total cases and severe cases. Then, we compared the modeled incidences with those observed in target series regarding their precision and accuracy. We also included the estimated incidence from sentinel surveillance as a reference for comparison. We estimated 3 models, model 1 is the basic model; model 2 is intermediate and adds positivity rate as a covariate; and model 3 is the final adjusted model, which adds SARS-CoV-2 predominant variants. The comparison between observed series and model 3 predictions can be observed in Figs. 3 and 4.

In Table 1, it is evident that the Pearson correlation between the total observed cases and the estimated cases through SARI sentinel surveillance at the national level, based on data from regions with surveillance information, is low ($$\rho =0.157$$). This is expected, as total cases encompass asymptomatic and mild cases, in addition to severe cases. Nevertheless, when incorporating variables such as positivity rate and the proportion of dominant COVID-19 variants into model 3, the correlation increases significantly ($$\rho =0.935$$). Regarding the series of severe cases, the correlation with SARI sentinel surveillance is initially high ($$\rho =0.892$$), but it experiences a notable increase in model 3 series ($$\rho =0.975$$). Similarly, the distance between estimated and observed cases decreases, both for total cases ($$DTW=66.596$$ to $$19.358)$$ and severe cases $$(DTW= 8.565$$ to $$0.547$$), this suggests a greater resemblance to the target series in model 3 series. At the regional level (Table S1), this trend persists, and the highest correlations ($$\rho >0.9$$) and smaller distances are predictably observed for model 3 ($$DT{W}_{total}\sim 20.0;DT{W}_{severe}<1.0$$) in regions with larger populations and in the most remote regions from the country's center as well ($$DT{W}_{severe}<0.9$$).

**Table 1:** Comparison of errors between incidence models for infections and hospitalizations for national series.

Outcome	Model	Cor	DTW	sMAPE	B/SE	MSE	95% CI coverage
Infections (total cases)	SARI rate (direct estimation)	0.157	66.596	1.312	− 65.237	47,627.479	0.006
	Model 1: basic	0.463	46.903	0.610	0.791	25,904.375	0.104
	Model 2: intermediate	0.702	34.984	0.423	0.680	16,014.177	0.195
	Model 3: final*	0.935	19.358	0.274	0.167	6945.675	0.279
	Model 3* (national series**)	0.934	22.403	0.315	0.379	8855.792	0.592
Hospitalizations (severe cases)	SARI rate (direct estimation)	0.892	8.565	1.222	5.470	317.201	0.000
	Model 1: basic	0.862	1.374	0.462	2.063	12.900	0.091
	Model 2: intermediate	0.846	1.198	0.358	1.802	11.031	0.097
	Model 3: final*	0.975	0.547	0.210	0.390	2.571	0.234
	Model 3* (national series**)	0.957	0.806	0.283	1.127	8.815	0.815

Cor: Pearson’s correlation; DTW: Dynamic Time Wrap distance; sMAPE = Symmetric mean absolute percentage error; B = Bias; SE: Standard Error; B/SE: Standardized bias; MSE: Mean Square Error; CI: Confidence Interval. *Proposed nowcasting model. **Incidence estimation for the entire national territory series; the other series correspond to the estimation in territories with sentinel centers for Severe Acute Respiratory Infections (SARI). For infections, Model 1 includes variables for time, sentinel surveillance incidence rate and hospitalization probability; Model 2 adds positivity rate; Model 3 adds proportion of COVID-19 dominant variants. For hospitalizations, Model 1 includes variables for time, sentinel surveillance incidence rate and sex; Model 2 adds positivity rate; Model 3 adds proportion of COVID-19 dominant variants.

Nationally, the accuracy for direct estimation from sentinel surveillance data is low (${sMAPE}_{total}= 1.312$;${sMAPE}_{severe}=1.222$, values closer to zero indicate higher accuracy) and it significantly improves with our model 3 (${sMAPE}_{total}= 0.274$; ${sMAPE}_{severe}= 0.21$) (Table 1). Standardized accuracy is lower in direct estimation ($B/{SE}_{total}=-65.237$;$B/{SE}_{severe}=5.47$, values closer to zero indicate higher accuracy) and higher in the model ($B/{SE}_{total}=0.167$;$B/{SE}_{severe}=0.39$). The relationship between precision and accuracy, measured as the mean squared errors, is also lower in the series estimated by model 3 (${{MSE}_{total}=\text{6,945.6}\text{; }MSE}_{severe}=$ $2.6$) and higher in the raw estimation from sentinel surveillance (${{MSE}_{total}=\text{47,627.5}\text{; }MSE}_{severe}=317.2$).

At the regional level, larger regions exhibit smaller errors, and in general we find that model 3 presents smaller errors. Both for total and severe cases, we observe the same weekly trend in errors as in their average values, with higher errors for the 1st (basic) model and lower errors for model 3. In general, the proposed model 3 shows stable errors throughout the study period, with a tendency to underestimate incidence during peak periods. Meanwhile, the simpler models exhibit fluctuating errors associated with maximum and minimum periods, with greater underestimation during maximum periods (Table 1).

**Figure 5:**
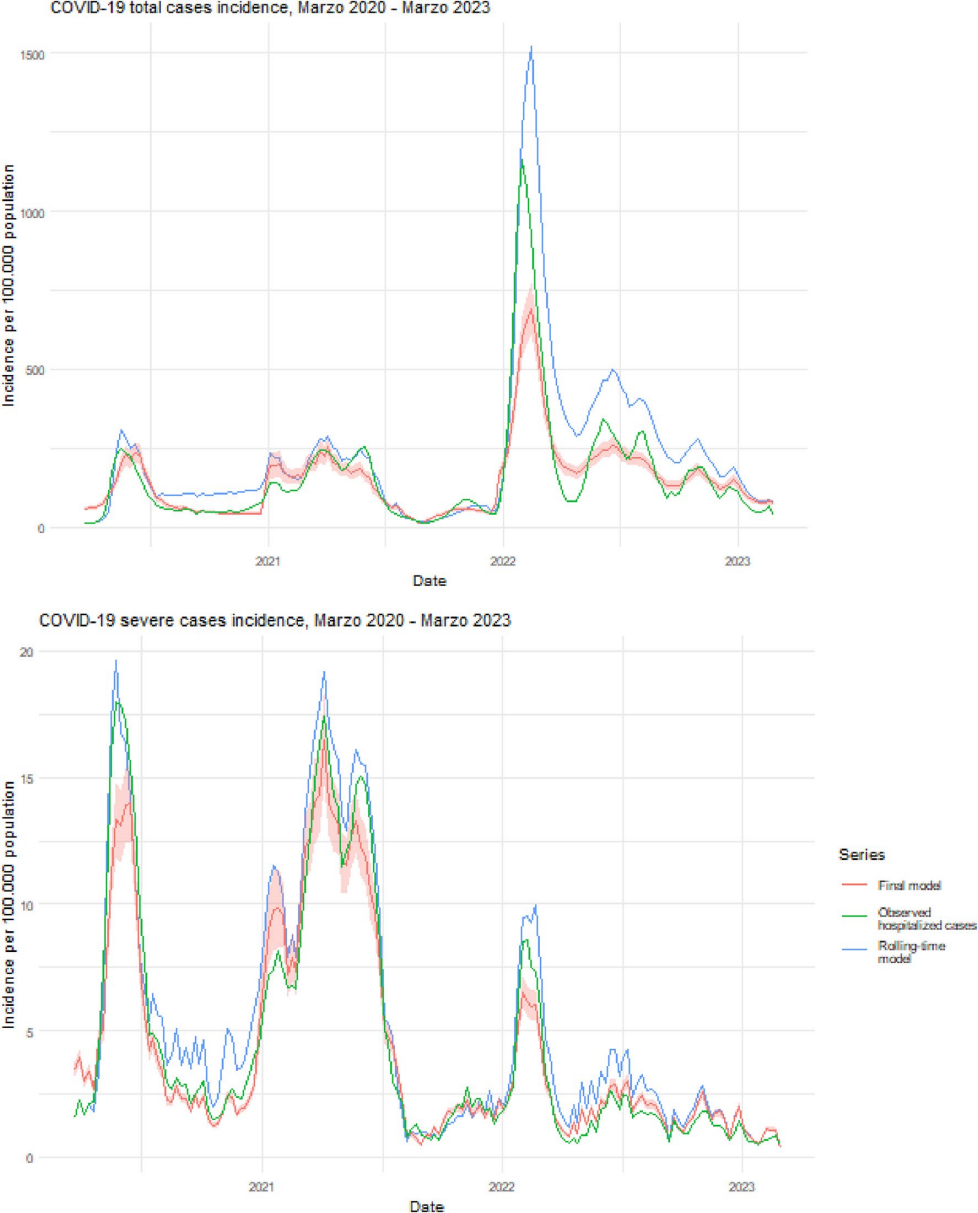
Comparison between Final model estimation and rolling-time nowcasting model. Incidence rate per 100,000 population for the national series of total cases (upper) and severe cases (lower) from March 1st 2020 to March 1st 2023. Blue lines represent the observed incidence rate of SARS-CoV-2 (with PCR or antigens test confirmation). Red lines represent the final model (model 3) estimation of incidence rate and red color bands around red lines represent the 95% confidence intervals of the fitted models. Green lines represent the rolling-time nowcasting model estimation of incidence rate.

Our model’s results (Table 2) for the incidence rate of total cases show a decrease in incidence over time (weeks) ($IRR=0.997, C{I}_{95\%}=0.996{-}0.997$). Furthermore, we found that an increase in the incidence of SARI sentinel and in the PCR and antigen tests positivity rate for SARS-CoV-2 is associated with an increase in the incidence rate ($IRR=1.002, C{I}_{95\%}=1.002{-}1.002$ for each unit increase in the rate per 100,000 inhabitants, and $IRR=1.047, C{I}_{95\%}=1.042{-}1.052$ for every 1% increase in positivity, respectively). To further assess the real-time applicability of our model and the impact of data availability delays, we performed additional analyses, considering a two-week lag for variant circulation data. Moreover, we conducted a running-time analysis to simulate the model's real-time performance, utilizing data available up to each time point (Fig. 5). This analysis reveals a commendable fit for severe cases, particularly at peak times, despite some overestimations during 2023 for total cases, probably due to substantial changes in COVID-19 testing strategies on the third pandemic year. Such changes in testing strategies modify the surveillance system and thus the parameters being measured, necessitating model recalibration. Nevertheless, on average errors are similar to those observed in the final model, stable during low-incidence periods and higher during peaks.

**Table 2:** Negative binomial response linear mixed model coefficients.

Infections (total cases)		Hospitalizations (severe cases)	
Variables	Coefficients	Variables	Coefficients
Fixed effects [Coef (95% CI)]			
Time (weeks)	0.997 (0.996–0.997)	Time (weeks)	0.998 (0.998–0.999)
Sentinel surveillance IR	1.002 (1.002–1.002)	Sentinel surveillance IR	1.002 (1.001–1.002)
Hospitalization probability	0.965 (0.963–0.968)	Sex (male)	1.241 (1.196–1.288)
Positivity rate	1.047 (1.042–1.052)	Positivity rate	1.045 (1.041–1.049)
Alpha	1.007 (0.993–1.020)	Alpha	1.005 (0.994–1.015)
B.1.1.348	1.013 (1.009–1.016)	B.1.1.348	1.010 (1.007–1.013)
B.1.1	1.010 (1.008–1.013)	B.1.1	1.005 (1.003–1.007)
Delta	1.015 (1.013–1.016)	Delta	1.005 (1.004–1.006)
Gamma	1.014 (1.012–1.017)	Gamma	1.012 (1.010–1.014)
Lambda	1.023 (1.018–1.028)	Lambda	1.018 (1.014–1.022)
Mu	0.966 (0.961–0.971)	Mu	0.966 (0.960–0.972)
N.4	1.015 (1.006–1.024)	N.4	1.011 (1.004–1.019)
Omicron	1.030 (1.028–1.032)	Omicron	1.007 (1.005–1.009)
Other	1.016 (1.011–1.020)	Other	1.007 (1.003–1.011)
Random effects [variance]			
Region:Age	0.141	Region:Age	0.590
Residuals	6.793	Residuals	16.969
ICC	0.020	ICC	0.034

Final model (model 3) coefficients. For other models, see supplementary tables. IRR: model coefficient expressed as incidence rate ratio; CI: confidence interval; IR: incidence rate; ICC: intraclass correlation coefficient.

The importance of the effect of circulating variants in model 3 is substantial, highlighting the importance of maintaining genomic surveillance as part of surveillance systems. All variants contribute significantly to the increase in incidence compared to the Wuhan SARS-CoV-2, except for the Alpha variant, with no difference ($IRR=1.007, C{I}_{95\%}=1.042{-}1.052$ for every 1% increase in the percentage of cases with this variant), and the Mu variant, that has a reverse relationship with incidence ($IRR=0.966, C{I}_{95\%}=0.961{-}0.971$). Within this first group, the Omicron ($IRR=1.030, C{I}_{95\%}=1.028{-}1.032$) and Lambda ($IRR=1.023, C{I}_{95\%}=1.018{-}1.028$) variants are strongly associated with an increase in incidence. We also observe that an increase in the percentage of cases requiring hospitalization is associated with a lower incidence rate ($IRR=0.965, C{I}_{95\%}=0.963{-}0.968$ for every 1% increase in the hospitalization probability). This happens because by controlling for sentinel surveillance incidence rate and circulating variants, the model already adjusts for changes in the severity of the virus; hence, the probability of hospitalization for cases acts as a proxy for the population immunity level against severe cases while the other population characteristics are kept constant.

Thus, for the severe cases model (Table 2), we find that time, incidence in SARI sentinel surveillance and positivity rate have a direct relationship with the response variable ($IRR=0.998$, $1.002$ and $1.045$, respectively). The same applies to those coefficients that are associated with variants, where most of them significantly contribute to an increase in incidence, with the exception of the Alpha variant, which shows no significance, and the Mu variant, which decreases it ($IRR=0.966, C{I}_{95\%}=0.960{-}0.972$). In this case, the variants that contribute the most to the increase in severe cases are different, with Lambda ($IRR=1.012, C{I}_{95\%}=1.010{-}1.014$) and Gamma ($IRR=1.018, C{I}_{95\%}=1.014{-}1.022$) having the greatest impact. Additionally, this model incorporates the sex variable since it has been described as a factor independently associated to the severity of COVID-19 cases; in this case, for male sex the rate of severe cases increases by 24.1%, compared to that for female sex ($C{I}_{95\%}=1.196{-}1.288$).

To assess the capability of the proposed models of adequately estimating the incidence rate of total and severe cases at the national level, including regions without SARI sentinel surveillance, we conducted the same comparisons. For infections, the model's national estimation is 4.62 million total cases ($C{I}_{95\%}=3.327$ millon $- 5.914$ millon), while for severe cases, the estimation is 240,000 severe events ($C{I}_{95\%}=$ 141,000–339,000). In both cases, the confidence interval captures the observed value of 4.894 million total cases and 206,000 observed hospitalizations (Table 3). The correlation between the estimated and observed series remains when regions without sentinel surveillance are added, meanwhile, the distance ($DTW$), accuracy ($sMAPE$), and standardized accuracy ($B/SE$) remain close in total cases, thus indicating that national estimates do not lose accuracy compared to estimates that only consider regions with SARI sentinel surveillance. On the other hand, the standardized accuracy in hospitalizations decreases, as does the relationship between accuracy and precision ($MSE$ increases) in total and severe cases, indicating a decrease in precision. Additionally, the confidence interval coverage increases for total cases and observed hospitalizations (59.2% and 81.5%, respectively). However, this increase in coverage comes with a loss of precision associated with its widening.

**Table 3:** Observed and predicted infections and hospitalizations at the national level per year.

Year	Infections (total cases)				Hospitalizations (severe cases)			
	Observed	Predicted	95% CI LB	95% CI UB	Observed	Predicted	95% CI LB	95% CI UB
2020	603,796	754,031	964,940	543,123	65,505	74,104	46,371	101,838
2021	1,145,505	1,181,030	1,532,500	829,561	95,133	109,366	60,182	158,551
2022	3,028,599	2,515,282	3,199,998	1,830,567	41,655	51,559	31,042	72,076
2023	116,428	170,123	216,482	123,763	3,525	4,986	3,359	6,614
Total	4,894,328	4,620,468	5,913,921	3,327,015	205,818	240,018	140,955	339,080

Final model (model 3) predictions. CI: Confidence Interval; LB: lower bound; UB: Upper bound.

The final models show a trend of decreasing infections and hospitalizations over time. This, along with the decrease in hospitalizations associated with the increase in infections, and the fact the strains that increase contagion compared to the Wuhan variant are not the same as those that increase hospitalizations, suggests an improvement in immunity. Therefore, in the contagion model, the proportion of hospitalizations serves as a proxy for the level of hybrid immunity in the analyzed population, acquired through vaccination and/or exposure to the virus, while the variants account for the virus' severity. These results underscore the importance of maintaining constant surveillance over the variants and their impact on public health.

## Discussion

The accuracy, precision, and timeliness of data obtained through sentinel surveillance for tracking epidemic outbreaks of ILI is an area that has been insufficiently studied. This reflects a weakness when evaluating the performance of systems and identifying improvement opportunities. In this study, we assessed the capacity of SARI sentinel surveillance to estimate the incidence of total and severe cases of an epidemic outbreak accurately and precisely. We used data collected during the COVID-19 pandemic in Chile as our study model, where we compared the universal surveillance systems and the population-based record of hospital beds with sentinel surveillance of respiratory diseases at both the national and subnational levels.

Our main results indicate that SARI sentinel surveillance tends to underestimate the incidence rate of total cases and overestimate severe cases compared to census or population-based records. While the literature in this area is limited, a similar study in Portugal found high correlations between COVID-19 cases and SARI surveillance data, however, it did not perform population-based incidence estimates^24^. Another study focusing on the estimation of severe cases from SARI surveillance in Germany also observed an overestimation of hospitalizations, something they attributed to deficiencies in the mandatory reporting system^25^. However, this explanation does not apply to our setting because the information on severe cases comes from the country's census-based hospitalizations (hospital bed records) rather than an episodic notification system, indicating the need to consider other factors in the explanation. One such factor could be the potential difference in the incidence rate between public and private healthcare facilities. SARI sentinel surveillance is primarily conducted in public hospitals, while the hospital census covers both types of facilities. Hence, the overestimation could be explained by a higher incidence of hospitalization in public facilities compared to private ones. Another factor could be the inclusion of patients who were hospitalized with a positive COVID-19 test but not necessarily for COVID-19-related complications, potentially skewing the perceived severity of cases. If this were significant, we would expect the bias to be towards underestimation rather than overestimation of severe cases. This underscores the importance of distinguishing between causes of hospitalization in future research.

Other studies that have evaluated sentinel surveillance during the COVID-19 pandemic have focused on ILI surveillance-based forecasting but have not attempted to improve estimates through nowcasting^26^. In this study, we corrected the estimates through statistical modeling of the incidence rate by complementing SARI sentinel surveillance with other routinely collected data. We used a generalized linear model with mixed effects and negative binomial response, which significantly improved the correlation, accuracy, and precision of the estimates. Furthermore, the integration of genomic surveillance data significantly enhances model predictions for COVID-19, reinforcing the importance of timely acquisition of such data and suggesting a promising avenue for monitoring influenza variants and reinforcing the model's broader applicability in infectious disease surveillance. This finding suggests that the incorporation of relatively simple modeling techniques can be a fundamental tool to complement traditional descriptive surveillance, generating more reliable and useful results for public health decision-making. Furthermore, our sensitivity analysis, leveraging data from 9 sentinel centers to estimate national incidence, including predictions for regions without such surveillance, underlines the adaptability of our model to address incomplete reporting. This confirms our nowcasting approach's potential to mitigate sentinel surveillance data challenges.

For decades, sentinel surveillance has prioritized information reports, choosing timeliness over their imprecision or biases. However, it is now possible to implement real-time nowcasting techniques. The incorporation of additional analyses demonstrates our model's adaptability and effectiveness in a real-time scenario, highlighting its potential utility for ongoing and future surveillance efforts. In addition to improving the accuracy and precision of estimates, these techniques can address delays in information reporting at a low cost^19,27^. Meanwhile, their statistical implementation is straightforward, ensuring that the timeliness of the estimates is suitable for use in monitoring and surveillance of ongoing outbreaks. This approach leads to better detection of ongoing epidemic outbreaks in their early phases and facilitates informed decision making in real time during public health crises, which can favor the timely implementation of control and preventive measures. To facilitate this process, the codes used in this study have been implemented using open-source software, require minimal computational resources, and are available for replication and use by government health agencies in various contexts (GitHub link).

Other studies have shown the effectiveness of nowcasting in assessing the epidemiological situation of COVID-19 by using different data sources, such as daily case notifications to correct for underreporting or reporting delay, and patterns detected in internet searches or interactions to estimate incidences. However, these studies rely on information made available due to additional resources allocated to address the pandemic or the extensive media coverage of it^19,27–29^. Our work focuses on revaluing the information collected by sentinel surveillance of respiratory diseases, which extends beyond the COVID-19 pandemic. This type of modeling can be applied to other emerging and re-emerging diseases if the data is collected under similar surveillance systems with analogous recording of the information. Systems that are consistent over time support the model's applicability across different pathogens. However, it is crucial to highlight that while the modeling strategy, including the model type and variables, is replicable, each model must be tailored to the specific data of its target population. Our model does not explicitly control for policy changes, but these variables can be introduced in dynamic scenarios to enhance model accuracy. Our approach underscores the utility and applicability of traditional sentinel surveillance in real-time decision-making, vis-a-vis with modern modelling techniques, significantly contributing to a more effective response to future epidemiological threats.

The information gathered during the COVID-19 pandemic in Chile not only provides valuable insights for addressing this disease but also serves as a case study for evaluating respiratory virus surveillance systems. As a model, it transcends the boundaries of a specific case, allowing for its application to various contexts and pathogens, such as seasonal influenza and those with pandemic potential. The results obtained can be extrapolated to other countries that maintain sentinel surveillance systems and have comparable data sources. However, it is important to note that changes in testing strategies represent changes in the surveillance system itself, thereby altering what is being measured. Consequently, any change in testing strategy necessitates recalibrating the model to accurately reflect the new measurement system. Additionally, thanks to the simplicity of the model used, this approach can be implemented in a wide range of epidemiological settings using open-source software, minimal data requirements, and basic computational capabilities, making it a versatile tool for the detection of emerging or circulating pathogens and for obtaining critical information for the effective implementation of public health measures.

However, it is essential to acknowledge the limitations of this study. Some regions included in the analysis began participating in SARI sentinel surveillance at later dates, which could have affected the precision of the estimates. Additionally, estimates for regions without SARI surveillance were based on assumptions that may not accurately reflect reality. While our models adjusted for various variables, there may be unaccounted or unmeasured factors that influence case incidence. Furthermore, it's worth noting that SARI sentinel surveillance provides information from severe cases, and implementing similar models on ILI sentinel surveillance could allow for even earlier outbreak detection capacities^30^. This couldn't be evaluated in this study due to the interruption of ILI surveillance during the first two years of the COVID-19 pandemic in Chile.

In conclusion, this study underscores the benefits of applying statistical nowcasting models in epidemiological surveillance. These models complement traditional descriptive surveillance and improve the accuracy and precision of population incidence estimates from existing data. Although primarily demonstrated with COVID-19 data, these approaches have the potential to be extended to other respiratory diseases, contributing to the surveillance of emerging or circulating pathogens and enhancing public health preparedness and response to respiratory diseases. This is vital for early detection of epidemic outbreaks and informed decision-making in public health.

## Methods

### Data

Through a request to the Ministry of Health, we obtained time series of anonymized data from epidemiological surveillance for the period between March 1, 2020 (EW10) and March 1, 2023 (EW09). The epidemiological surveillance series consist of the count of reported cases at the epidemiological week level, stratified by sex, age, and geographical unit (municipality and region) from both the universal surveillance of COVID-19 (which includes asymptomatic, ILI and SARI cases) and the hospital-based sentinel surveillance of SARI. The case definition of ILI is characterized by a fever of 38 °C or higher, cough and the apparition of symptoms within 10 previous days to medical consultation, while the definition of SARI includes respiratory distress and hospitalization in addition to those symptoms^31^. Additionally, we obtained weekly counts of hospitalizations for COVID-19, stratified by sex, age and region, from the centralized bed management unit's census record. We supplemented this information with weekly national-level data on the predominant SARS-CoV-2 variant^32^, the positivity rate of COVID-19 tests^33^ and population projection by municipality data for the same period^34^.

To harmonize the datasets, we worked with geographical units at the regional level, determined by the region of the healthcare facility, and grouped ages into twenty-year intervals. This choice was based on the observation of similar epidemiological dynamics within each age group and notable differences between groups. We limited our analysis to patients up to 80 years due to the limitations of population projections. We grouped case counts in universal and SARI sentinel surveillance by symptoms` onset week. For asymptomatic cases, we used the date of sample collection. For hospitalizations, we used the date of hospital admission. Data necessary to replicate the analyses is available in the paper's repository (https://github.com/sfloresalvarado/sari-sentinel-eval).

### Analysis

For this work we considered two response variables: the incidence rate of COVID-19 infections and the incidence rate of hospitalizations for COVID-19. For both variables we had census information, and for the modeling we used the count of infections or hospitalizations with an offset for the population (for population size estimation in sentinel centers). We fitted generalized linear models with mixed effects, negative binomial response, and a log-link function. For all models, the mixed-effects component is a slope for age nested within regions. The basic model (model 1) for the incidence of cases included fixed effects for time, the weekly incidence rate of SARI sentinel surveillance, and the proportion of COVID-19 cases that require hospitalization. The intermediate model (model 2) adds positivity rate (the proportion of SARS-CoV-2 tests with a positive result), and the final model (model 3) adds variables indicating the variant share over time of the 10 predominant SARS-CoV-2 variants during the 2020–2023 period. For hospitalizations incidence, the fixed effects for the basic model include the variables of time, weekly incidence rate of SARI sentinel surveillance and sex; the intermediate and final models add positivity rate and predominant variants, respectively. Preliminary analyses indicated that the incidence of COVID-19 infections is similar between both sexes, but the incidence of hospitalizations differs, in line with published literature^35^. Hence, this variable was not included in the models for infections, but it was included in the models for hospitalizations. Also, we did not consider the proportion of vaccinated individuals because its effect is already captured by the SARI incidence and the proportion of cases that require a hospitalization, therefore, the inclusion of the vaccination coverage did not change the estimates in alternative models. Additionally, we adjusted a rolling-time model to evaluate the performance of the model as if it was performed on real time, at each time point from ${t}_{i}=4$ to ${t}_{i}=159$, considering data from $t = \{{t}_{1}, \dots , {t}_{i}\}$.

We evaluated the series estimated by the models by comparing them to the national and regional population series of infections and hospitalizations, as appropriate. At the weekly level, we estimated the symmetric mean absolute percentage error ($sMAPE$), standardized bias ($B/SE$) and mean squared error ($MSE$). $sMAPE$ values vary between 0 and 2; a $sMAPE$ close to zero indicates an accurate estimation and low error relative to the parameter; its increasing value towards positive values indicates overestimations compared to those observed, while negative values indicate underestimations. $B/SE$ corresponds to accuracy standardized by precision; when its absolute value is close to one, it indicates that the bias magnitude is equal to its variability; if it is greater than one, the estimator is inaccurate relative to its precision, and if it is less than 1, precision is greater than accuracy. $MSE$ provides a general measure of the relationship between accuracy and precision of the estimates, where lower values indicate more accurate and precise estimations.

At the complete series level, we estimated the Pearson correlation, which measures the linear relationship between the estimated and observed series; a high correlation suggests that the estimates closely follow the observations. Additionally, we calculated the dynamic time warping ($DTW$) distance between the two series, which quantifies their similarity, so a higher value increases the distance. We also assessed the coverage of the 95% confidence interval, indicating how often it contains the true parameter value. Along with this, we evaluated the mean of each of the weekly indicators. Since SARI sentinel centers are placed in only 8 regions, only these were considered for modeling and initial evaluation, at the national and regional levels. Furthermore, we made national-level estimates considering all 16 regions and compared them with the complete national series of infections and hospitalizations. For regions without sentinel surveillance, the SARI rate of the geographically nearest region with surveillance was assigned, stratifying by week and age for infections and adding sex for hospitalizations.

The supplementary material provides additional information on the calculation of the variables included in the models and on the distance, accuracy and precision measures used to compare the target and modeled series.

### Supplementary Information

Supplementary Information.

## Data Availability

The data that support the findings of this study are available in a GitHub repository, https://github.com/sfloresalvarado/sari-sentinel-eval. A fraction of the data is not publicly available but can be accessed from the Ministry of Health upon reasonable request, https://transparencia.redsalud.gob.cl/transparencia/public/ssp/solicitud_informacion.html.

## References

[1] McCloskey B, Dar O, Zumla A, Heymann DL (2014). Emerging infectious diseases and pandemic potential: Status quo and reducing risk of global spread. Lancet Infect. Dis..

[2] Bauch CT, Oraby T (2013). Assessing the pandemic potential of MERS-CoV. Lancet.

[3] Zocchi E, Terrazzano G (2021). COVID-19: Why not learn from the past?. Front. Med..

[4] Ibrahim NK (2020). Epidemiologic surveillance for controlling Covid-19 pandemic: Types, challenges and implications. J. Infect. Public Health.

[5] Gupta S, Gupta T, Gupta N (2022). Global respiratory virus surveillance: Strengths, gaps, and way forward. Int. J. Infect. Dis..

[6] World Health Organization. End-to-end integration of SARS-CoV-2 and influenza sentinel surveillance: Revised interim guidance [Internet] (2022). https://www.who.int/publications-detail-redirect/WHO-2019-nCoV-Integrated_sentinel_surveillance-2022.1 [cited 2022 Jun 1].

[7] Choi BCK (2012). The past, present, and future of public health surveillance. Scientifica (Cairo).

[8] World Health Organization. Surveillance in emergencies [Internet]. https://www.who.int/emergencies/surveillance [cited 2022 Apr 29].

[9] Murray, J. & Cohen, A. L. Infectious disease surveillance. In *International Encyclopedia of Public Health [Internet]* 2nd edn (ed. Quah, S. R.) 222–229 (Academic Press, Paris, 2017) https://www.sciencedirect.com/science/article/pii/B9780128036785005178 [cited 2022 May 6].

[10] Porta M (2014). A Dictionary of Epidemiology.

[11] Global Influenza Surveillance and Response System (GISRS) [Internet]. https://www.who.int/initiatives/global-influenza-surveillance-and-response-system [cited 2022 May 6].

[12] Shedura VJ, Hussein AK, Nyanga SK, Kamori D, Mchau GJ (2023). Evaluation of the influenza-like illness sentinel surveillance system: A national perspective in Tanzania from January to December 2019. PLoS One.

[13] Rakotoarisoa A, Randrianasolo L, Tempia S, Guillebaud J, Razanajatovo N, Randriamampionona L (2017). Evaluation of the influenza sentinel surveillance system in Madagascar, 2009–2014. Bull. World Health Organ..

[14] Ribeiro IG, Sanchez MN (2020). Evaluation of the severe acute respiratory syndrome (SARS) surveillance system, with emphasis on influenza, Brazil, 2014–2016. Epidemiol. Serv. Saude.

[15] Rosenthal M, Anderson K, Tengelsen L, Carter K, Hahn C, Ball C (2017). Evaluation of sampling recommendations from the influenza virologic surveillance right size roadmap for Idaho. JMIR Public Health Surveill..

[16] Suhail Y, Afzal J, Kshitiz (2021). Incorporating and addressing testing bias within estimates of epidemic dynamics for SARS-CoV-2. BMC Med. Res. Methodol..

[17] Ricoca Peixoto V, Nunes C, Abrantes A (2020). Epidemic surveillance of Covid-19: Considering uncertainty and under-ascertainment. Port. J. Public Health.

[18] Banbura, M., Giannone, D. & Reichlin, L. Nowcasting [Internet] (2010). https://papers.ssrn.com/abstract=1717887 [cited 2023 Oct 13].

[19] Greene SK, McGough SF, Culp GM, Graf LE, Lipsitch M, Menzies NA (2021). Nowcasting for real-time COVID-19 tracking in New York City: An evaluation using reportable disease data from early in the pandemic. JMIR Public Health Surveill..

[20] Wu JT, Leung K, Lam TTY, Ni MY, Wong CKH, Peiris JSM (2021). Nowcasting epidemics of novel pathogens: Lessons from COVID-19. Nat. Med..

[21] Subsecretaría de Salud Pública, Ministerio de Salud, Gobierno de Chie. Aprueba el reglamento sobre notificación de enfermedades transmisibles de declaración obligatoria y su vigilancia. Decreto, N°7/2019 (2020).

[22] Taramasco C, Rimassa C, Romo J, Zavando A, Bravo R (2022). Epidemiological surveillance in COVID-19 pandemic: EPIVIGILA system. Medwave.

[23] Depto. de Epidemiología, Ministerio de Salud, Gobierno de Chile. Visualización Interactiva de Influenza [Internet]. http://epi.minsal.cl/Vigilancia_influenza/ [cited 2023 May 25].

[24] Torres AR, Gómez V, Kislaya I, Rodrigues AP, Fernandes Tavares M, Pereira AC (2023). Monitoring COVID-19 and influenza: The added value of a severe acute respiratory infection surveillance system in Portugal. Can. J. Infect. Dis. Med. Microbiol..

[25] Tolksdorf K, Haas W, Schuler E, Wieler LH, Schilling J, Hamouda O (2022). ICD-10 based syndromic surveillance enables robust estimation of burden of severe COVID-19 requiring hospitalization and intensive care treatment [Internet]. medRxiv.

[26] Glatman-Freedman A, Gur-Arie L, Sefty H, Kaufman Z, Bromberg M, Dichtiar R (2022). The impact of SARS-CoV-2 on respiratory syndromic and sentinel surveillance in Israel, 2020: A new perspective on established systems. Eurosurveillance.

[27] Jersakova R, Lomax J, Hetherington J, Lehmann B, Nicholson G, Briers M (2022). Bayesian imputation of COVID-19 positive test counts for nowcasting under reporting lag. J. R. Stat. Soc. Ser. C Appl. Stat..

[28] Kogan NE, Clemente L, Liautaud P, Kaashoek J, Link NB, Nguyen AT (2021). An early warning approach to monitor COVID-19 activity with multiple digital traces in near real time. Sci. Adv..

[29] Mavragani A (2020). Tracking COVID-19 in Europe: Infodemiology approach. JMIR Public Health Surveill..

[30] Toh KB, Runge M, Richardson RA, Hladish TJ, Gerardin J (2023). Design of effective outpatient sentinel surveillance for COVID-19 decision-making: A modeling study. BMC Infect. Dis..

[31] Surveillance and monitoring [Internet]. https://www.who.int/teams/global-influenza-programme/surveillance-and-monitoring [cited 2023 May 25].

[32] Instituto de Salud Pública, Ministerio de Salud, Gobierno de Chile. Variantes SARS-CoV-2 [Internet]. https://vigilancia.ispch.gob.cl/app/varcovid [cited 2023 Aug 7].

[33] Ministerio de Ciencia T Conocimiento e Innovación. GitHub—MinCiencia/Datos-COVID19 [Internet] (2020). https://github.com/MinCiencia/Datos-COVID19 [cited 2020 Oct 14].

[34] Instituto Nacional de Estadísticas, Gobierno de Chile. Default. Proyecciones de Población. http://www.ine.gob.cl/estadisticas/sociales/demografia-y-vitales/proyecciones-de-poblacion [cited 2023 Aug 7].

[35] Mukherjee S, Pahan K (2021). Is COVID-19 gender-sensitive?. J. Neuroimmune Pharmacol..

