# Supplementary material for: Nowcasting methods to improve the performance of respiratory sentinel surveillance: lessons from the COVID-19 pandemic
Source: Sci Rep. 2024 May 31;14:12582. doi: 10.1038/s41598-024-62965-5 (PMC11143190; doi:10.1038/s41598-024-62965-5)
Supplement: Supplementary file 1 — Supplementary Information. [file 41598_2024_62965_MOESM1_ESM.docx]

Supplementary material

Contents

Supplementary Tables 2

Table S1: Comparison of errors between incidence models for infections and hospitalizations for regional series. 2

Table S2a: Negative binomial response linear mixed model coefficients for the final and intermediate models for infections incidence rate. 4

Table S2b: Negative binomial response linear mixed model coefficients for the final and intermediate models for hospitalizations incidence rate. 5

Supplementary Figures 6

Figure S1: Incidence rate of COVID-19 total cases vs. SARI sentinel surveillance estimates adjusted by hospitalization rate 6

Figure S2: Incidence rate of COVID-19 total cases vs. SARI sentinel surveillance estimates by sex and age 7

Figure S3: Incidence rate of COVID-19 hospitalizations vs. SARI sentinel surveillance estimates by sex and age 8

Figure S4: Assessment of errors in the incidence estimation, March 2020 - March 2023 9

Figure S5: Bland-Altman plot for the incidence estimation, March 2020 - March 2023 10

Figure S6: Comparison between selected final model and rolling-time nowcasting model 11

Figure S7: Comparison between selected final model and alternative final model incorporating 2 weeks’ delay on variant circulation reporting. 12

Supplementary methods 13

Methods S1: Estimation of the catchment population size of the SARI sentinel centers geographic area of influence 13

Methods S2: Comparison between selected final model and alternative final model incorporating COVID-19 second and booster vaccination doses coverage within the population. 15

Methods S3: Variables and comparison methods formulas and definitions 16

# Supplementary Tables

### Table S1: Comparison of errors between incidence models for infections and hospitalizations for regional series.

| Model | Level | Infections (total cases) | | | | | | Hospitalizations (severe cases) | | | | | |
| --- | --- | --- | --- | --- | --- | --- | --- | --- | --- | --- | --- | --- | --- |
|  |  | Cor | DTW | sMAPE | B/SE | MSE | 95% CI coverage | Cor | DTW | sMAPE | B/SE | MSE | 95% CI coverage |
| SARI rate (direct estimation) | Chile | 0.157 | 66.596 | 1.312 | -65.237 | 47627.479 | 0.006 | 0.892 | 8.565 | 1.222 | 5.47 | 317.201 | 0 |
|  | Metropolitana | 0.36 | 54.809 | 0.664 | -12.425 | 32687.017 | 0.156 | 0.908 | 21.597 | 1.399 | 2.362 | 1889.005 | 0.097 |
|  | Tarapacá | 0.366 | 70.576 | 0.767 | -38.984 | 83873.563 | 0.028 | 0.811 | 1.545 | 0.83 | 0.492 | 34.101 | 0.724 |
|  | Antofagasta | 0.357 | 89.089 | 0.762 | -48.539 | 79017.553 | 0 | 0.72 | 1.751 | 0.886 | 0.42 | 29.508 | 0.747 |
|  | Valparaíso | 0.024 | 70.125 | 0.68 | -32.688 | 57448.865 | 0.097 | 0.899 | 6.617 | 1.088 | 1.14 | 313.604 | 0.435 |
|  | Bío Bío | -0.035 | 78.526 | 0.628 | -29.537 | 89004.456 | 0.338 | 0.822 | 34.14 | 1.315 | 2.81 | 3522.528 | 0.179 |
|  | La Araucanía | 0.29 | 73.725 | 0.75 | -11.918 | 87099.737 | 0.182 | 0.773 | 20.699 | 1.501 | 1.727 | 1408.669 | 0.180 |
|  | Los Lagos | 0.087 | 59.851 | 0.746 | -9.764 | 84497.65 | 0.448 | 0.726 | 58.193 | 1.664 | 2.451 | 10020.092 | 0.059 |
|  | Magallanes | 0.668 | 79.543 | 0.66 | -10.527 | 88341.379 | 0.337 | 0.856 | 30.99 | 1.717 | 0.874 | 4539.584 | 0.439 |
| Model 1: basic | Chile | 0.463 | 46.903 | 0.61 | 0.791 | 25904.375 | 0.104 | 0.862 | 1.374 | 0.462 | 2.063 | 12.9 | 0.091 |
|  | Metropolitana | 0.43 | 45.651 | 0.664 | 0.868 | 20431.068 | 0.078 | 0.898 | 1.673 | 0.499 | 1.762 | 24.486 | 0.078 |
|  | Tarapacá | 0.636 | 55.345 | 0.767 | 2.38 | 47349.456 | 0.103 | 0.861 | 1.425 | 0.734 | 2.997 | 14.965 | 0.106 |
|  | Antofagasta | 0.456 | 71.782 | 0.762 | 1.035 | 41010.362 | 0.15 | 0.876 | 1.094 | 0.636 | 1.411 | 7.882 | 0.132 |
|  | Valparaíso | 0.46 | 51.269 | 0.68 | 0.9 | 31496.551 | 0.091 | 0.826 | 1.284 | 0.586 | 2.303 | 12.27 | 0.082 |
|  | Bío Bío | 0.431 | 55.978 | 0.628 | 0.663 | 56404.424 | 0.156 | 0.73 | 1.329 | 0.463 | 1.363 | 22.695 | 0.172 |
|  | La Araucanía | 0.46 | 61.488 | 0.75 | 1.061 | 57048.345 | 0.11 | 0.797 | 1.35 | 0.607 | 1.833 | 13.041 | 0.128 |
|  | Los Lagos | 0.29 | 64.714 | 0.746 | 1.31 | 68535.561 | 0.182 | 0.594 | 3.834 | 0.639 | 2.004 | 137.689 | 0.158 |
|  | Magallanes | 0.746 | 66.768 | 0.66 | 1.751 | 36742.876 | 0.143 | 0.898 | 0.814 | 0.45 | 0.535 | 3.377 | 0.512 |
| Model 2: intermediate | Chile | 0.702 | 34.984 | 0.423 | 0.68 | 16014.177 | 0.195 | 0.846 | 1.198 | 0.358 | 1.802 | 11.031 | 0.097 |
|  | Metropolitana | 0.696 | 28.976 | 0.457 | 0.616 | 12797.822 | 0.117 | 0.904 | 1.18 | 0.367 | 1.171 | 12.874 | 0.234 |
|  | Tarapacá | 0.352 | 65.029 | 0.705 | 2.388 | 63696.199 | 0.076 | 0.846 | 1.462 | 0.707 | 3.232 | 15.139 | 0.098 |
|  | Antofagasta | 0.619 | 48.277 | 0.585 | 0.959 | 30555.374 | 0.106 | 0.846 | 1.087 | 0.595 | 1.393 | 8.377 | 0.187 |
|  | Valparaíso | 0.655 | 43.596 | 0.551 | 0.61 | 22087.058 | 0.097 | 0.746 | 1.327 | 0.517 | 2.108 | 14.518 | 0.075 |
|  | Bío Bío | 0.679 | 55.768 | 0.52 | 0.602 | 36819.712 | 0.182 | 0.651 | 1.945 | 0.45 | 1.19 | 32.261 | 0.113 |
|  | La Araucanía | 0.718 | 57.446 | 0.667 | 0.799 | 36038.233 | 0.045 | 0.743 | 1.345 | 0.56 | 1.68 | 15.562 | 0.143 |
|  | Los Lagos | 0.577 | 63.692 | 0.657 | 1.51 | 49552.276 | 0.156 | 0.531 | 4.497 | 0.586 | 1.707 | 178.407 | 0.171 |
|  | Magallanes | 0.718 | 61.21 | 0.566 | 1.879 | 41424.779 | 0.173 | 0.949 | 0.674 | 0.389 | 0.205 | 2.214 | 0.634 |
| Model 3: final | Chile | 0.935 | 19.358 | 0.274 | 0.167 | 6945.675 | 0.279 | 0.975 | 0.547 | 0.21 | 0.39 | 2.571 | 0.234 |
|  | Metropolitana | 0.901 | 20.096 | 0.314 | 0.451 | 6053.157 | 0.26 | 0.946 | 0.924 | 0.279 | 0.691 | 11.989 | 0.162 |
|  | Tarapacá | 0.657 | 35.333 | 0.509 | 1.734 | 37342.276 | 0.241 | 0.899 | 0.814 | 0.529 | 1.836 | 8.303 | 0.187 |
|  | Antofagasta | 0.756 | 35.799 | 0.403 | 0.869 | 22617.571 | 0.327 | 0.911 | 0.794 | 0.428 | 1.472 | 4.867 | 0.253 |
|  | Valparaíso | 0.915 | 28.189 | 0.391 | 0.164 | 12101.851 | 0.201 | 0.966 | 0.591 | 0.289 | 0.976 | 2.71 | 0.259 |
|  | Bío Bío | 0.913 | 38.403 | 0.455 | 0.248 | 20293.537 | 0.182 | 0.861 | 1.295 | 0.382 | 0.912 | 14.915 | 0.172 |
|  | La Araucanía | 0.903 | 39.82 | 0.55 | 0.471 | 22973.667 | 0.175 | 0.935 | 0.657 | 0.432 | -0.434 | 4.526 | 0.241 |
|  | Los Lagos | 0.833 | 45.931 | 0.625 | 0.897 | 31358.867 | 0.136 | 0.801 | 1.844 | 0.478 | 0.459 | 24.24 | 0.184 |
|  | Magallanes | 0.851 | 36.595 | 0.394 | 0.64 | 21383.479 | 0.265 | 0.930 | 0.631 | 0.366 | 0.381 | 2.267 | 0.537 |
| Model 3: final (national series*) | Chile | 0.934 | 22.403 | 0.315 | 0.379 | 8855.792 | 0.592 | 0.957 | 0.806 | 0.283 | 1.127 | 8.815 | 0.815 |

### Table S2a: Negative binomial response linear mixed model coefficients for the final and intermediate models for infections incidence rate.

| **Variables** | **Model 0: null** | **Model 1: basic** | **Model 2: intermedite** | **Model 3: final** |
| --- | --- | --- | --- | --- |
| Fixed effects [IRR (95%CI)] | | | | |
| (Intercept) | 0.002 (0.002 - 0.002) | 0 (0 - 0) | 0 (0 – 0.001) | 1.205e+23 (1.245e+21 - 1.169e+25) |
| Time (weeks) |  | 1.001 (1 - 1.001) | 1 (1 - 1) | 0.997 (0.996 - 0.997) |
| Sentinel surveillance IR |  | 1.003 (1.003 - 1.004) | 1.003 (1.003 - 1.003) | 1.002 (1.002 - 1.002) |
| Hospitalization probability |  | 0.954 (0.952 - 0.957) | 0.959 (0.956 - 0.961) | 0.965 (0.963 - 0.968) |
| Positivity |  |  | 1.090 (1.085 - 1.094) | 1.047 (1.042 - 1.052) |
| Alpha |  |  |  | 1.007 (0.993 - 1.020) |
| B.1.1.348 |  |  |  | 1.013 (1.009 - 1.016) |
| B.1.1 |  |  |  | 1.010 (1.008 - 1.013) |
| Delta |  |  |  | 1.015 (1.013 - 1.016) |
| Gamma |  |  |  | 1.014 (1.012 - 1.017) |
| Lambda |  |  |  | 1.023 (1.018 - 1.028) |
| Mu |  |  |  | 0.966 (0.961 - 0.971) |
| N.4 |  |  |  | 1.015 (1.006 - 1.024) |
| Omicron |  |  |  | 1.030 (1.028 - 1.032) |
| Otras |  |  |  | 1.016 (1.011 - 1.020) |
| Random effects [Variance] | | | | |
| Region:Age | 0.099 | 0.141 | 0.162 | 0.141 |
| Residuals | 1.022 | 1.773 | 3.483 | 6.793 |
| ICC | 0.088 | 0.074 | 0.044 | 0.02 |
| Adjustment | | | | |
| AIC | 58525.934 | 57196.949 | 55610.766 | 54135.07 |
| BIC | 58544.821 | 57234.722 | 55654.835 | 54242.095 |
| logLik | -29259.967 | -28592.474 | -27798.383 | -27050.535 |
| Deviance | 58519.934 | 57184.949 | 55596.766 | 54101.07 |

IRR: model coefficient expressed as incidence rate ratio; CI: confidence interval; IR: incidence rate; ICC: intraclass correlation coefficient; AIC: Akaike information criteria; BIC: Bayesian information criteria; logLik: log-likelihood. In bold: statistically significant coefficients for the explanatory variable.

### Table S2b: Negative binomial response linear mixed model coefficients for the final and intermediate models for hospitalizations incidence rate.

| **Variables** | **Model 0: null** | **Model 1: basic** | **Model 2: intermedite** | **Model 3: final** |
| --- | --- | --- | --- | --- |
| Fixed effects [IRR (95%CI)] | | | | |
| (Intercept) | 0 (0 - 0) | 4,905.621 (610.307 – 39,431.155) | 20,834.936 (2,464.102 – 176,167.436) | 1,362,249,353.786 (12,787,980.244 – 145,114,651,925.174) |
| Time (weeks) |  | 0.999 (0.999 - 0.999) | 0.999 (0.999 - 0.999) | 0.998 (0.998 - 0.999) |
| Sentinel surveillance IR |  | 1.003 (1.002 - 1.003) | 1.003 (1.002 - 1.003) | 1.002 (1.001 - 1.002) |
| Sex (Male) |  | 1.181 (1.127 - 1.239) | 1.192 (1.140 - 1.247) | 1.241 (1.196 - 1.288) |
| Positivity |  |  | 1.040 (1.036 - 1.044) | 1.045 (1.041 - 1.049) |
| Alpha |  |  |  | 1.005 (0.994 - 1.015) |
| B.1.1.348 |  |  |  | 1.010 (1.007 - 1.013) |
| B.1.1 |  |  |  | 1.005 (1.003 - 1.007) |
| Delta |  |  |  | 1.005 (1.004 - 1.006) |
| Gamma |  |  |  | 1.012 (1.010 - 1.014) |
| Lambda |  |  |  | 1.018 (1.014 - 1.022) |
| Mu |  |  |  | 0.966 (0.960 - 0.972) |
| N.4 |  |  |  | 1.011 (1.004 - 1.019) |
| Omicron |  |  |  | 1.007 (1.005 - 1.009) |
| Otras |  |  |  | 1.007 (1.003 - 1.011) |
| Random effects [Variance] | | | | |
| Region:Age | 0.878 | 0.588 | 0.603 | 0.59 |
| Residuals | 2.521 | 4.815 | 6.071 | 16.969 |
| ICC | 0.258 | 0.109 | 0.09 | 0.034 |
| Adjustment | | | | |
| AIC | 30512.552 | 29361.176 | 28931.7 | 27442.071 |
| BIC | 30531.36 | 29398.792 | 28975.584 | 27548.648 |
| logLik | -15253.276 | -14674.588 | -14458.85 | -13704.036 |
| Deviance | 30506.552 | 29349.176 | 28917.7 | 27408.071 |

IRR: model coefficient expressed as incidence rate ratio; IR: incidence rate; CI: confidence interval; ICC: intraclass correlation coefficient; AIC: Akaike information criteria; BIC: Bayesian information criteria; logLik: log-likelihood. In bold: statistically significant coefficients for the explanatory variable.

# Supplementary Figures

### Figure S1: Incidence rate of COVID-19 total cases vs. SARI sentinel surveillance estimates adjusted by hospitalization rate


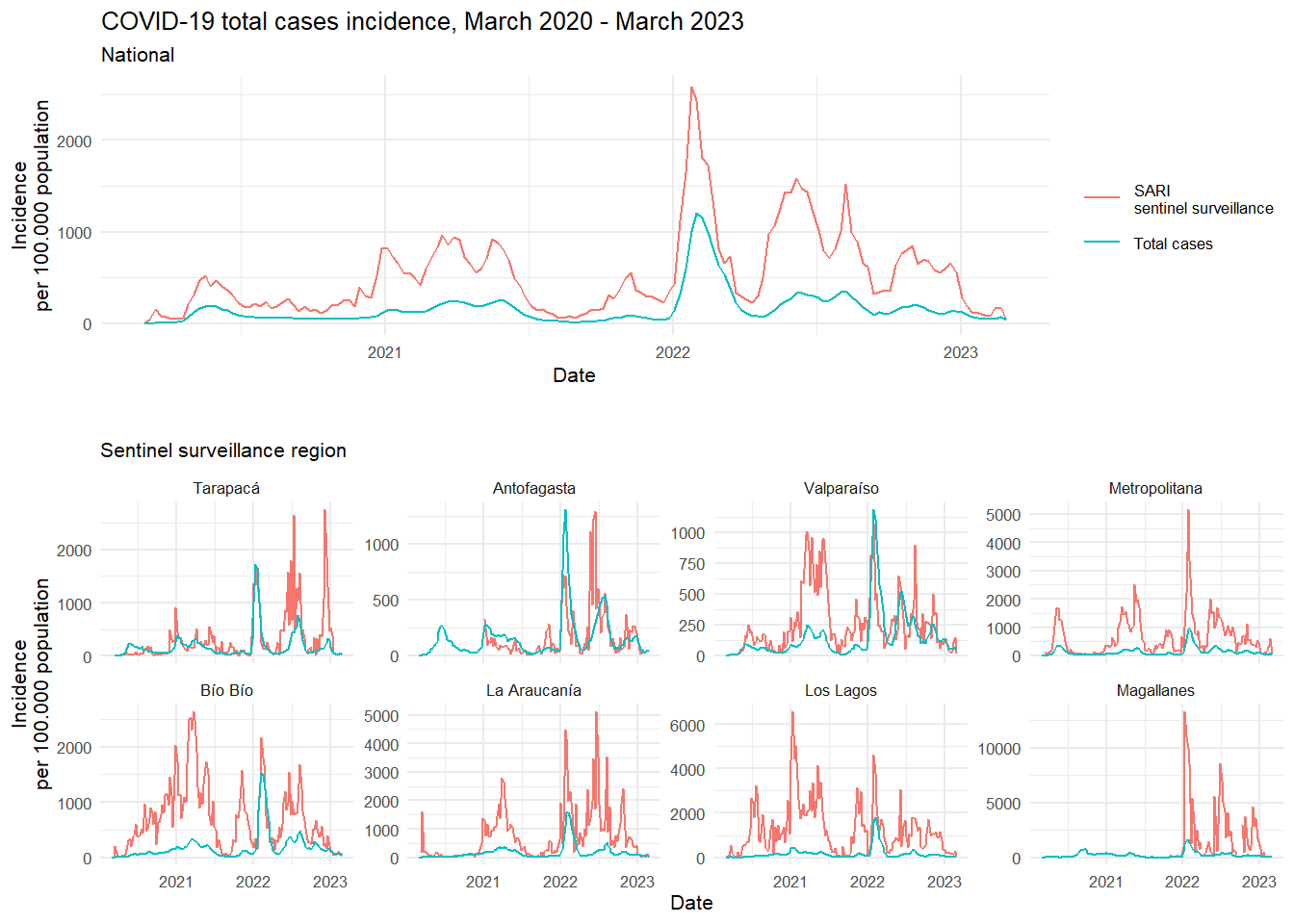


The SARI (Severe Acute Respiratory Infections) sentinel surveillance estimation has been adjusted using the hospitalization rate to estimate the total number of cases, as opposed to solely hospitalized cases.

### Figure S2: Incidence rate of COVID-19 total cases vs. SARI sentinel surveillance estimates by sex and age


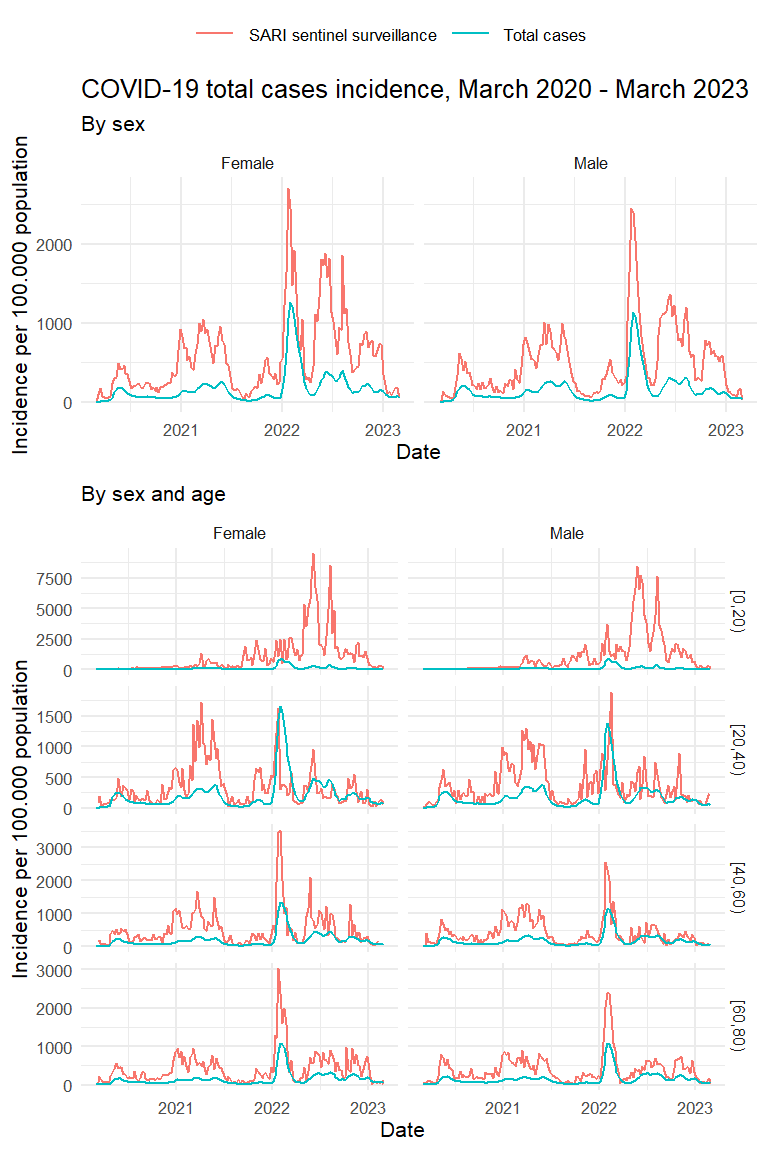


The SARI (Severe Acute Respiratory Infections) sentinel surveillance estimation has been adjusted using the hospitalization rate to estimate the total number of cases, as opposed to solely hospitalized cases.

### Figure S3: Incidence rate of COVID-19 hospitalizations vs. SARI sentinel surveillance estimates by sex and age


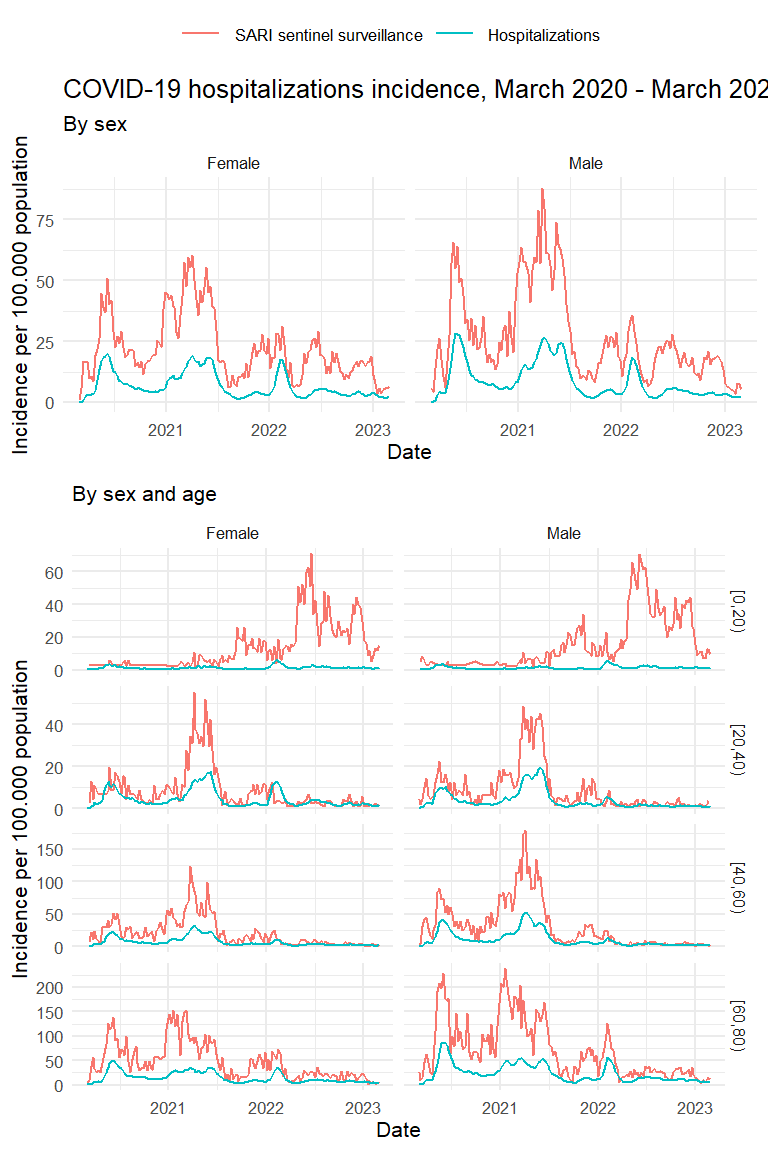


### Figure S4: Assessment of errors in the incidence estimation, March 2020 - March 2023

| 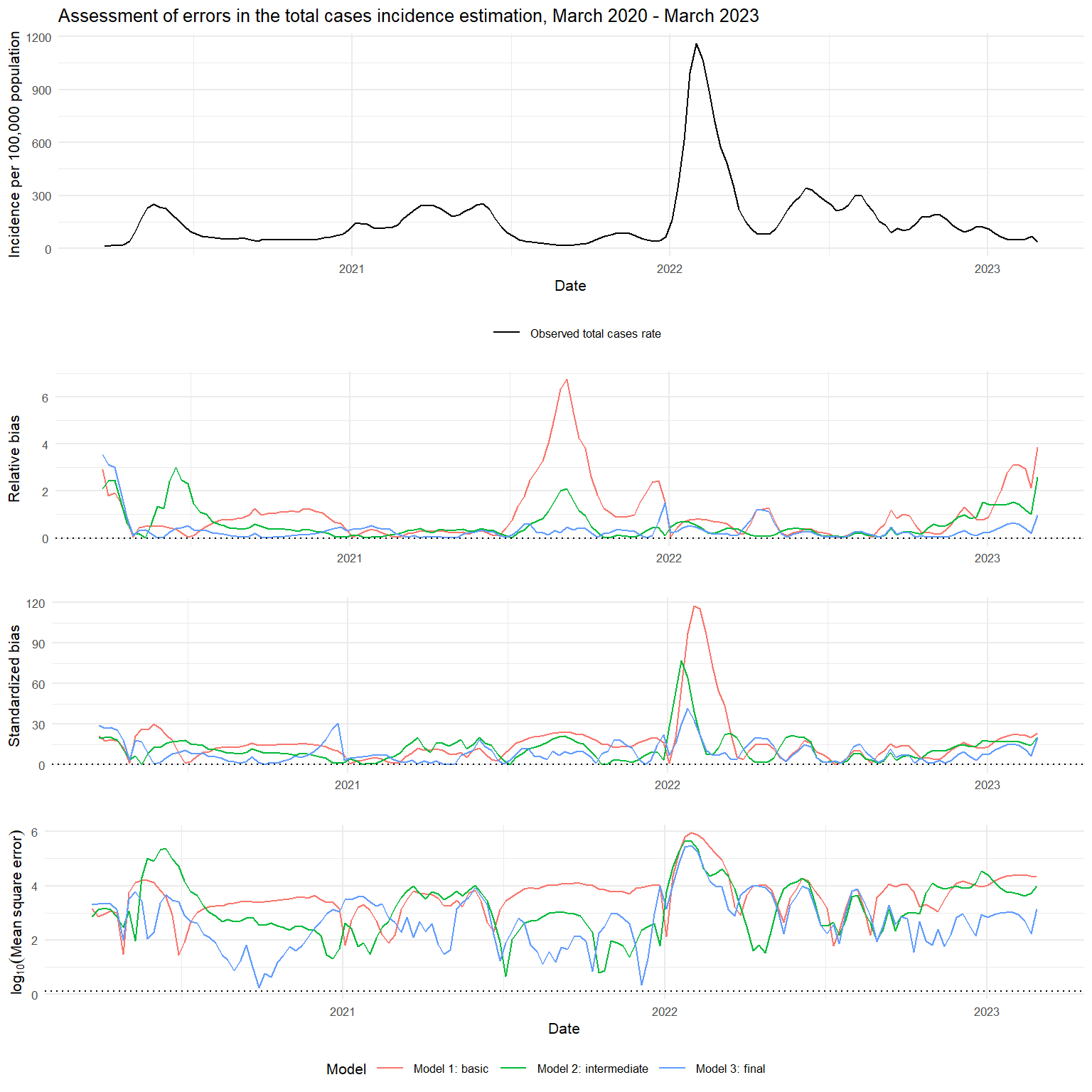 | 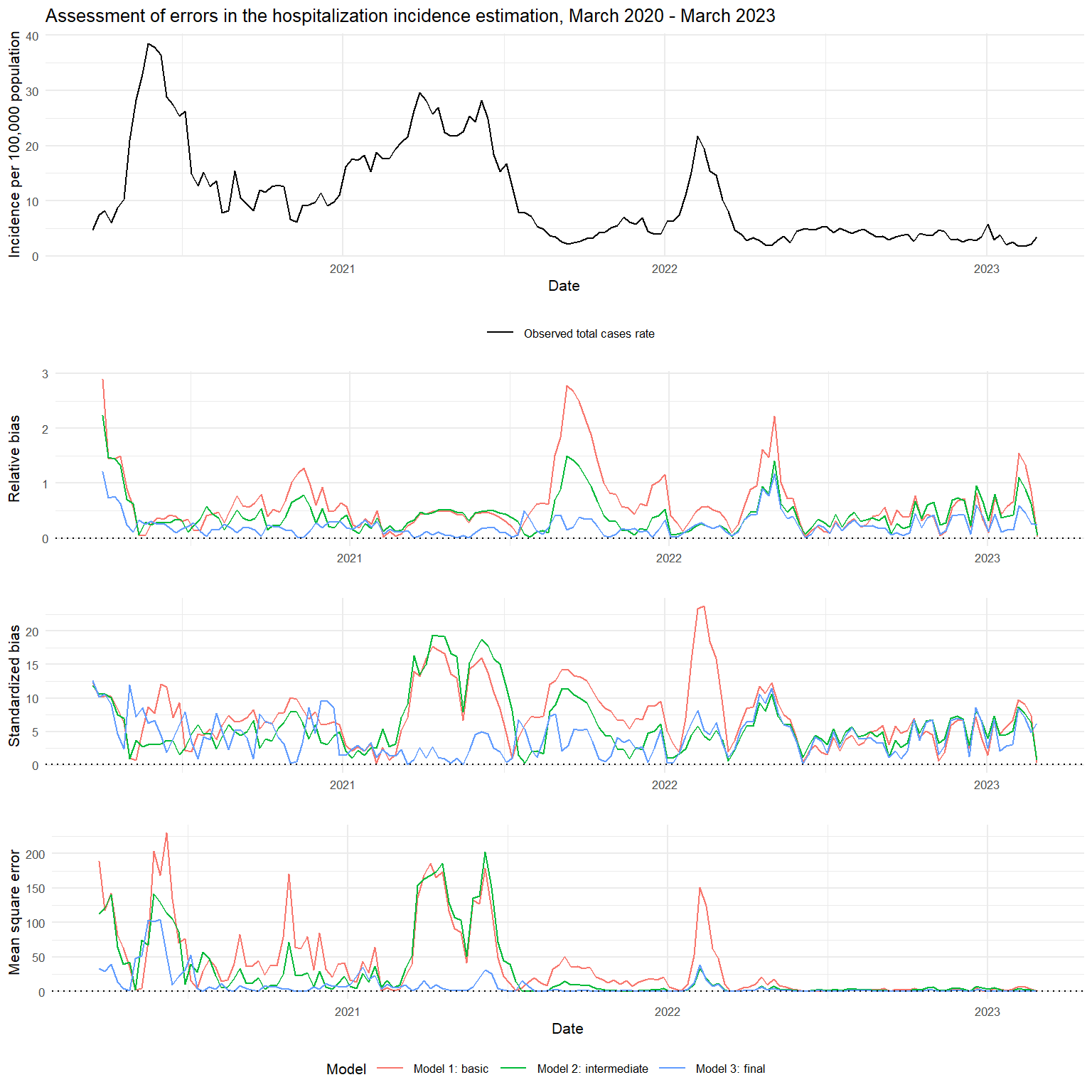 |
| --- | --- |

### Figure S5: Bland-Altman plot for the incidence estimation, March 2020 - March 2023

| 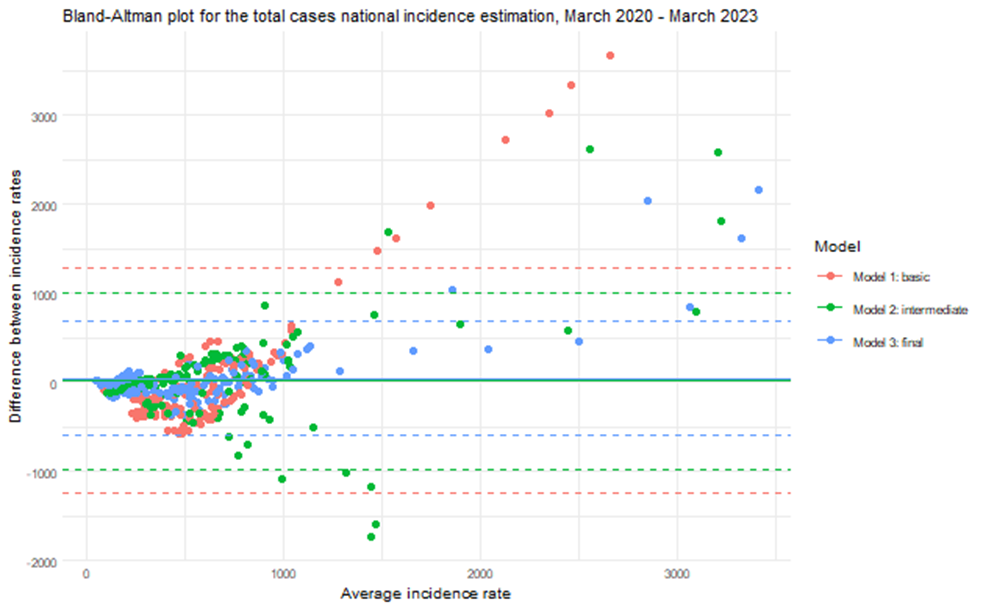 | 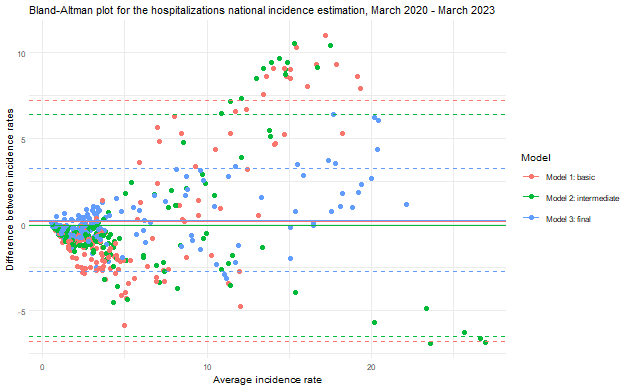 |
| --- | --- |

### Figure S6: Comparison between selected final model and rolling-time nowcasting model

|  | Infections (total cases) | Hospitalizations (severe cases) |  |
| --- | --- | --- | --- |
| Complete rolling-time nowcasting models | 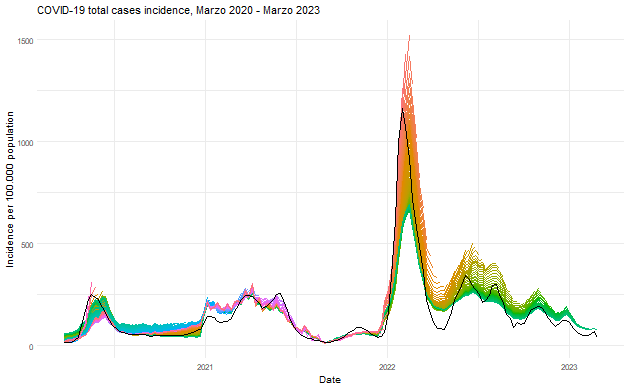 | 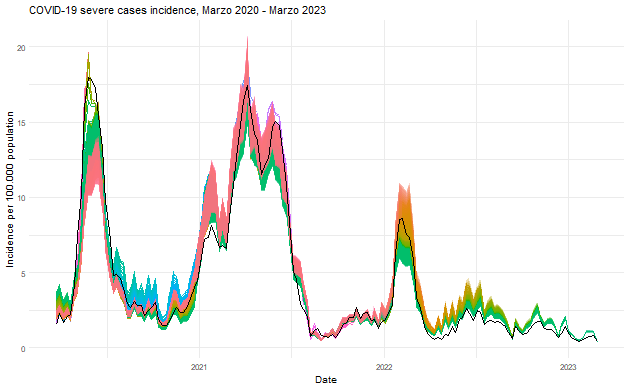 | In black: Final model adjusted with retrospective data available in week 159. In colors: Model adjusted with data from week=1 to week=t, t = {1, …, 159}. |
| Rolling-time model series constructed from the last point of each nowcasting model | **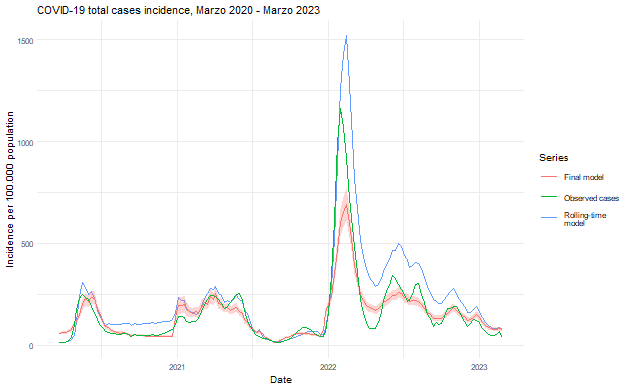** | 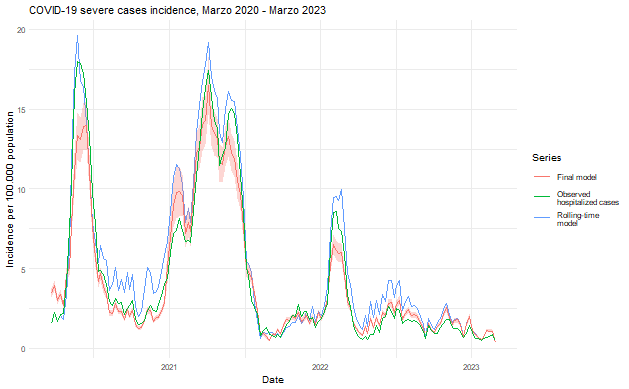 | Final model (red) was adjusted with retrospective data available in week 159. Rolling-time model (blue) series shows the last point of the model adjusted in week t, from t=4 to t=159.Both were compared to the observed series of cases and severe cases, respectively (green). |

### Figure S7: Comparison between selected final model and alternative final model incorporating 2 weeks’ delay on variant circulation reporting.

| Infections (total cases) | Hospitalizations (severe cases) |
| --- | --- |
| 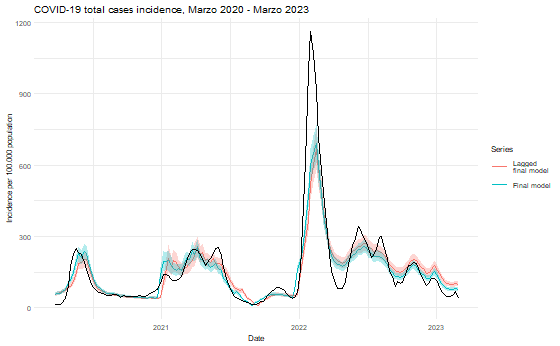 | 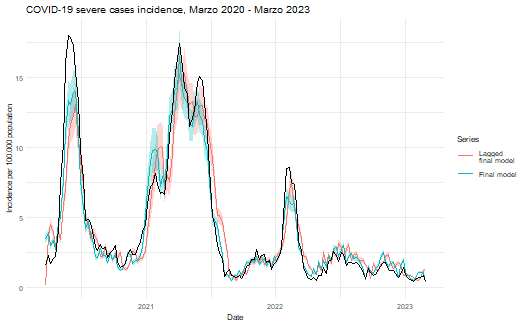 |

Black line represents the observed total or severe cases, respectively.

# Supplementary methods

### Methods S1: Estimation of the catchment population size of the SARI sentinel centers geographic area of influence

The accurate estimation of incidence rates for Severe Acute Respiratory Infections (SARI) using sentinel surveillance data necessitates the determination of an appropriate population denominator. This population represents the catchment area served by the sentinel centers, from which the reported cases are drawn. In this study, we applied the method based on the geographic area of influence, which uses the proportion of cases received by the sentinel center compared to other hospitals in neighboring areas to estimate population incidence. The applied method was based on the "Methodological Guide for the Estimation of the Disease Burden due to Severe Acute Respiratory Infections (SARI)" developed by PAHO/MINSAL (2017)^[[1]](#footnote-1)^ to adapt the WHO`s guidelines (2015)^[[2]](#footnote-2)^ to the Chilean context.

We used the following data sources to apply this method:

- Annual population projection by municipality, sex, and age (2020-2023)
- FONASA (Fondo Nacional de Salud) beneficiary population by year, municipality, sex, and age (2020-2022)
- Hospital discharges for respiratory causes J09-J22 and U07 (2020-2022)
- SARI sentinel surveillance (2020-2023)

We performed two separate catchment population size calculations. The first one considered the national population, and the second one considered the population covered by FONASA. For the first case, since population estimates are only performed up to the age of 80, we removed observations of individuals over 80 years old from the hospital discharge and SARI sentinel surveillance databases.

Municipality Case Allocation: We first identified municipalities with SARI case records and calculated the case counts ($n_{SARI}$) and population by region ($r$), municipality ($m$), establishment ($e$), sex ($s$), and age group ($a$) in the whole period.

$$n_{SARI}=\sum a_{r,m,e, s,a}$$

Where $x=1$ for SARI cases. Municipalities served by multiple sentinel centers were assigned to the center with the majority of cases of that municipality.

Case Proportion Calculation: For each center, we calculated the proportion of cases from each municipality relative to the total cases at the center.

$$\hat{p}_{e}=\frac{\sum a_{SARI,m,e}}{\sum a_{SARI,e}}$$

Where $p_{c}$ is the proportion of cases in a sentinel center (establishment) that are catched by one municipality.

Calculating Hospital Catchment Proportions: For municipalities served by more than one SARI sentinel center, a decision was made to attribute the municipality to the center where it constituted a larger proportion of cases. The proportions of cases from each municipality relative to the total cases handled by the center were calculated and sorted, with a cumulative sum used to retain municipalities contributing to up to 80% of the center's cases, so $\sum\hat{p}_{e}\leq0.8$.

Adjusting Population Figures: The catchment population estimates were adjusted by comparing the number of SARI cases ($n_{SARI}$) managed by the sentinel centers with the number of hospital discharges for respiratory causes ($n_{EH}$) in the same region, to account for the share of hospitalizations each center covers within its catchment area. This step involves aggregating data to the level of sentinel centers and calculating proportions of SARI cases to total respiratory hospital discharges, making further adjustments for years 2022 and 2023 based on observed trends and projections.

$$p_{m}=\frac{n_{SARIr,e}}{n_{EH,r}}$$

In cases where we observed atypical or implausible values in this proportion, we replaced the count of hospital discharges with the count from the previous year. In addition, since information on hospital discharges for 2023 is not yet available, we used those for 2022, weighted by the proportion of SARI cases up to the analysis cutoff date relative to the total cases in 2022.

Final Population Estimates for Sentinel Centers: Finally, we weighted the population projections or the total population or FONASA covered population ($N$) by year ($y$), region, municipality, sex, and age, by this proportion.

$$\hat{N}_{catchment,y,r,m,s,a}=N_{y,r,m,s,a}\times p_{m}$$

For the analyses, we used the catchment populations estimated using the census population projection. For each year, the populations for each SARI sentinel center calculated are as follows:

| **Facility** | **2020** | **2021** | **2022** | **2023** |
| --- | --- | --- | --- | --- |
| Hospital Clínico Regional Dr. Guillermo Grant Benavente (Concepción) | 31.864 | 44.953 | 114.147 | 181.239 |
| Hospital de Magallanes Dr. Lautaro Navarro Avaria | - | 25.684 | 7.234 | 9.846 |
| Hospital de Puerto Montt | 9.693 | 14.970 | 59.233 | 54.026 |
| Hospital Dr. Ernesto Torres Galdames (Iquique) | 80.467 | 106.719 | 103.864 | 106.091 |
| Hospital Dr. Gustavo Fricke (Viña del Mar) | 71.763 | 38.627 | 88.475 | 125.388 |
| Hospital Dr. Hernán Henríquez Aravena (Temuco) | 29.863 | 30.162 | 28.964 | 88.131 |
| Hospital Dr. Leonardo Guzmán (Antofagasta) | - | 63.909 | 100.789 | 155.352 |
| Hospital Militar de Santiago | 27.121 | 13.010 | 40.298 | 28.843 |
| Hospital San Juan de Dios (Santiago) | 32.052 | 24.664 | 19.058 | 56.557 |
| Total | 282.823 | 362.698 | 562.062 | 805.473 |

### Methods S2: Comparison between selected final model and alternative final model incorporating COVID-19 second and booster vaccination doses coverage within the population.


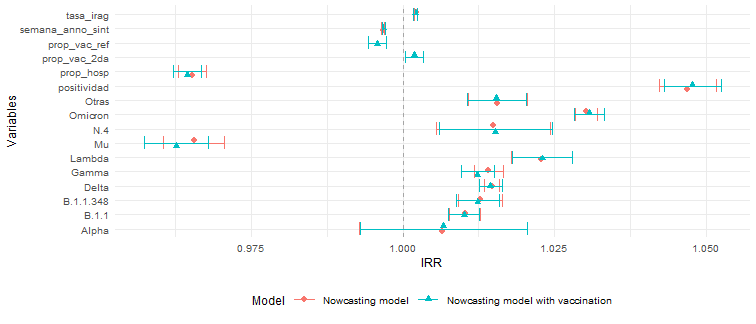


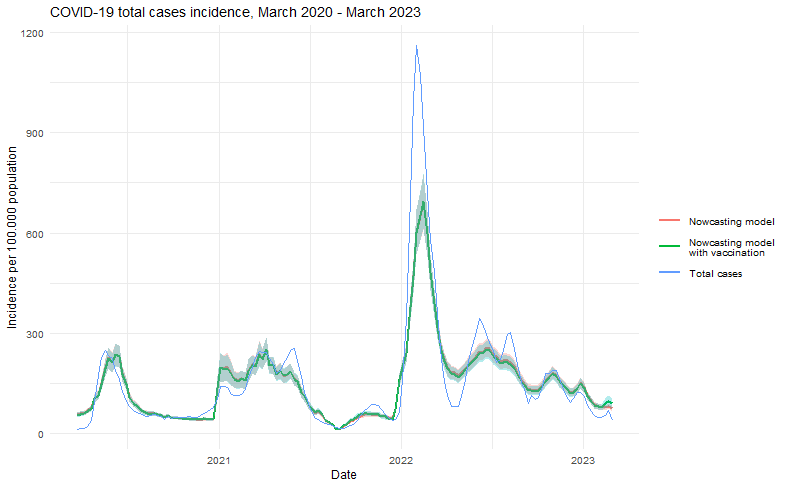


### Methods S3: Variables and comparison methods formulas and definitions

**Epidemiological variables**

| **Level** | **Variables** | **Formula** |
| --- | --- | --- |
| Week, region, sex, age | Total cases incidence rate | $\frac{new infections}{population}*100.000$ |
|  | Severe cases incidence rate | $\frac{new hospitalizations}{population}*100.000$ |
|  | Sentinel surveillance incidence rate | $IR_{SARI}=\frac{new SARI sentinel surveillance cases}{SARI sentinel surveillance population\text{*}}*100.000$ |
|  | Sentinel surveillance incidence rate adjusted by hospitalization rate | $\frac{IR_{SARI}}{hospitalization rate}$ |
|  | Hospitalization rate | $\frac{new hospitalizations}{new infections}*100$ |
| Week, region | Positivity rate | $\frac{positive PCR tests}{total PCR tests}*100$ |
| Week | SARS-CoV-2 variant proportion | $\frac{variant samples}{total samples}$ |
|  | 2^nd^ dose vaccination rate | $\frac{\sum vaccinated population}{total population}*100$ |
|  | Reinforcement vaccination rate |  |

*For SARI sentinel surveillance population estimation, see Methods S1.

**Comparison metrics**

| **Level** | **Comparison metrics** | **Formula / R library::function** |
| --- | --- | --- |
| Week | Bias ($B_{i}$) | $\hat{\theta_{i}}-\theta_{i}$ |
|  | Relative bias ($B_{ri}$) | $\frac{B_{i}}{\theta_{i}}$ |
|  | Standardized bias ($\left[ \frac{B}{SE} \right]_{i}$) | $\frac{B_{i}}{SE_{i}}$ |
|  | Mean squared error ($MSE_{i}$) | $B_{i}^{2}+SE_{i}^{2}$ |
| Full series | Mean relative bias ($B_{r}$) | $\frac{1}{n}\sum B_{ri}$ |
|  | Mean standardized bias ($\frac{B}{SE}$) | $\frac{1}{n}\sum\left[ \frac{B}{SE} \right]_{i}$ |
|  | Mean mean squared error ($MSE$) | $\frac{1}{n}\sum{MSE}_{i}$ |
|  | Symmetric mean absolute percentage error ($sMAPE$) | $\frac{1}{n}\sum\frac{\vert\theta_{i}-\hat{\theta}_{i}\vert}{(\vert\theta_{i}\vert+\vert\hat{\theta}_{i}\vert)/2}$ |
|  | Pearson’s correlation ($Cor$) | stats::cor() |
|  | Dynamic time warping ($DTW$) | dtw::dtw() |
|  | 95% Confidence interval coverage | $\frac{1}{n}\sum I_{i}, I_{i}= \left\{ \begin{aligned} 1 if \hat{\theta}\in IC95\% \\ 0 \sim\end{aligned} \right.$ |

$SE$: standard error; $\theta$: target series (parameter); $\theta_{i}$: target observation; $\hat{\theta}$: predicted series (estimator); $\hat{\theta}_{i}$: predicted observation; $n$: número de semanas

1. De la Fuente, Felipe. (2017). Guía metodológica para la estimación de carga de enfermedad por Infecciones respiratorias Agudas Graves (IRAG). Santiago, Chile: Organización Panamericana de la Salud / Ministerio de Salud de Chile. [↑](#footnote-ref-1)
2. WHO Global Influenza Progamme. (2015). A manual for estimating disease burden associated with seasonal influenza. Ginebra. [↑](#footnote-ref-2)
